# Blaberidae (Blattodea) of Madre de Dios, Peru and a key to Epilamprinae s. s.

**DOI:** 10.3897/zookeys.1281.178573

**Published:** 2026-06-04

**Authors:** Katharine Vanker, Emmy Fiorella Medina-Espinoza, Johanna Schwartz, Jared Martin, Dominic A. Evangelista

**Affiliations:** 1 School of Integrative Biology, University of Illinois Urbana-Champaign, 505 S Goodwin Ave., Urbana, Illinois, 61801, USA Museo de Historia Natural, Universidad Nacional Mayor de San Marcos Lima Peru; 2 Program in Ecology, Evolution and Conservation, School of Integrative Biology, University of Illinois Urbana-Champaign, 505 S Goodwin Ave., Urbana, Illinois, 61801, USA School of Integrative Biology, University of Illinois Urbana-Champaign Urbana United States of America; 3 Departamento de Entomología, Museo de Historia Natural, Universidad Nacional Mayor de San Marcos, Av. Arenales 1256, Jesús María, Lima, Peru Program in Ecology, Evolution and Conservation, School of Integrative Biology, University of Illinois Urbana-Champaign Urbana United States of America; 4 Entomology Department, School of Integrative Biology, University of Illinois Urbana-Champaign, 505 S Goodwin Ave., Urbana, Illinois, 61801, USA Entomology Department, School of Integrative Biology, University of Illinois Urbana-Champaign Urbana United States of America; 5 Entomology Department, Smithsonian National Museum of Natural History, 1000 Madison Drive NW, Washington, D.C. 20560, USA Entomology Department, Smithsonian National Museum of Natural History Washington United States of America

**Keywords:** Amazon, alpha taxonomy, Blaberinae, Blaberoidea, Blattaria, cyber taxonomy, tergal gland, Zetoborinae

## Abstract

Significant progress has been made in the cockroach faunistics of some South American countries (i.e., Brazil, Ecuador, Colombia, and the Guiana shield) during the last 25 years while the Peruvian fauna has remained almost entirely understudied. The Peruvian Amazonia is recognized as having one of the most biodiverse and endemic faunas in the world. Yet, this is not reflected in the relatively depauperate species list of cockroaches from Peru. We partially rectify this knowledge gap by providing a thorough report on the Blaberidae of the department of Madre De Dios and an updated checklist of Blaberidae of Peru. To accomplish this, we conducted a partial revision of Epilamprinae*sensu stricto*, accompanied with a diagnostic key and visual guide that distinguishes all Peruvian genera, and most other genera, based on male sexual morphology. This includes the description of one new species group, five newly described species of *Epilampra* (*E. yompori*, *E. homage*, *E. wandpero*, *E. sofia*, *E. paititi*), and three potential new species not described due to taxonomic uncertainty (*E. conferta* “placa”, *E. conferta* “barra”, E. sp. cf. azteca ). Additionally, we described a new species, *Lanxoblatta patriciae***sp. nov**., and the first new species of *Phoetalia*, *P. amigo***sp. nov**. in 160 years. We also provided new country records of three existing taxa, one of them (*Hyporhicnoda humilior* Hebard, 1933) for the whole South America. Overall, we provided a checklist to the 30 species of Blaberidae in Madre De Dios, bringing the count for all of Peru up to 39.

## Introduction

Peru constitutes one of the largest gaps in Blattodea knowledge in both the Amazonia and the Americas. After Brazil, Peru holds the second largest proportion of Amazonia (13%). Yet, the Peruvian Amazonia has more hotspots of species richness and endemism than Brazil (Mittermeier et al. 2000; Orme et al. 2005; Ceballos and Ehrlich 2006), primarily attributable to the geographical heterogeneity present on the eastern flank of the Andes (Lamas 1997). Despite this, Peru has comparatively few cockroach species known, and has a similar known richness to much smaller comparable countries (Peru has 109 spp. of non-termite Blattodea; Bolivia, 40; Venezuela, 78; Colombia, 109; Ecuador, 114 (Vidlička 2013); Guyana, 105 (Evangelista et al. 2016); Suriname, 136 (Evangelista et al. 2015); French Guiana, 163 (Evangelista et al. 2019); Brazil, 650 (Pellens and Grandcolas 2008; Hopkins and Beccaloni 2024; Deng et al. 2026). This is almost certainly a product of under sampling.

While a few publications have looked at a handful of Peruvian cockroaches (Shelford 1912; Albuquerque 1964) and other publications included taxonomic descriptions and records of species from the country (Rehn 1916; Hebard 1921, 1926; Rehn 1930), the cockroaches of Peru have never been comprehensively studied. The current study is part of a larger comprehensive survey of the cockroaches of the department of Madre De Dios.

Since 1994, Madre de Dios holds the designation of Biodiversity Capital of Peru, according to Law 26311/19941 (Congreso de la República del Perú (1994)). Currently, ~ 45% of its territory harbors 25 protected areas – 19 of which are privately managed (SERNANP 2025). Madre de Dios features 12 ecosystems, the most common by proportion of land cover being: low hill forest (i.e, non-flooded dissected terrain, with moderately sloping hills ranging from 20 to 80 meters in height; 62% of land cover), non-flooded terrace forest (11%), and flooded alluvial forest (11%) (MINAM 2018). This variety of habitats, jointly with its complex hydrogeomorphology (Hamilton et al. 2007), underpins the region’s high diversity: up to 128 trees species (Ter Steege et al. 2023), 114 amphibians (May et al. 2008), and ~600 fish (Amazon Fish Project; (Jezequel et al. 2020). Our preliminary data suggest that Madre De Dios is on par with French Guiana in terms of total cockroach biodiversity (Grandcolas 1994; Evangelista et al. 2019), with an estimated total richness of ~150 species – including ~ 22 species of Blaberidae that we have observed.

We report only Blaberidae species in this paper, with descriptions for new species, some revised diagnoses for existing taxa, and images of some taxa that are taxonomically unchanged but are lacking habitus images in existing taxonomic tools. While only two species of *Epilampra* Burmeister, 1838 were previously reported from Peru, we report nine species, including five newly described. Adding literature records and iNaturalist observations, we estimate there are about 32 total species of Blaberidae in Madre De Dios.

## Materials and methods

### Field methods

We collected adult Blaberidae in five different sites in the department of Madre de Dios, Peru: Los Amigos Biological Station, Puerto Maldonado (Kapievi Ecovillage and nearby areas), Ecoaventuras Amazónicas, Finca Las Piedras Research Station, and Kawsay Biological Station. The first three sites were sampled during July 2021, and the last two during June–July 2024. We employed hand collection, baited pitfall traps, and light traps as collection methods. Hand collection was performed mainly at night, usually between 17:00 h and 23:00 h, and individuals were killed in 70% ethanol. Pitfall traps consisted of plastic cups placed on the ground and baited with fermented drinks or rotten fish (Evangelista et al. 2015; Velez-Bravo and Daza 2021). In the case of fermented drinks, lager beer has previously been used as an effective bait (Evangelista et al. 2017) so we used this in addition to testing the efficacy of red wine. In Los Amigos and Puerto Maldonado, the three types of baited pitfall traps were used; while in Las Piedras and Kawsay, we only used fish. Traps remained active for three days in each locality. In fish-baited traps, the fish were placed above the traps using a cheesecloth and a stick. For light trapping, LED lights (white and ultraviolet) bulbs were suspended on a white cloth. These traps were set up from ~19:00 to ~22:00 hrs in each locality, except for Puerto Maldonado and Ecoaventuras Amazónicas, where light trapping was not employed. Hand collection was the most effective method to capture Blaberidae species (250 specimens), followed by fish baited pitfalls (33 specimens), and light trapping (31 specimens). Fermented-drink baited pitfalls traps were ineffective to attract Blaberidae specimens (eight specimens). To preserve color, some individuals were pinned after being initially killed in 70% ethanol. However, most collected individuals were preserved in 70–90% ethanol (higher concentration for larger bodied individuals) in whirl-pack bags for the duration of the field expedition and then frozen at -20C in the lab before being pinned and labelled.

### Morphological analysis and taxonomic practices

Specimens were visualized under Leica S9D or Nikon SMZ1270 stereomicroscopes with FlexCamC1 and PrimeCam Intervision SMZ12 cameras, respectively. Large specimens were imaged with a Canon EOS9 camera and a Canon 180 mm macro lens. When dry, selected specimens were photographed in front of an 18% grey card (except for *Epilampra paititi* sp. nov., and *E. wandpero* sp. nov.) for habitus representation. Images were manually post-edited to adjust color warmth, contrast, and brightness to best replicate the look in the lab under white light. Following Roth (1970a, 1970b, 1970c, 1970d), a traditional protocol for genitalia preparation was followed using 20–25% NaOH. Specimens’ abdominal tip was digested for 4–8 hours, or until soft tissues were largely cleared (Roth 1970a, 1970b, 1970c, 1970d). Focus stacks were assembled in Zerene Stacker v. 1.04.

Genital morphological terminology used is primarily based on to Anisyutkin (2016), the most recent work to treat these taxa, but comparisons to Roth (1970d); and Klass (1995) are given in Table 1. Most of the useful taxonomic literature on Epilamprinae (e.g., Roth 1970d) utilizes terminology consistent with McKittrick (1964). However, Anisyutkin (2016) mostly utilized Klass (1997)’s terminology. Klass (1997) built much of his system on top of McKittrick (1964), improving it with more detailed consideration of structures (including both hard and soft-tissues, as well as musculature), incorporating research revising side-homologies (Mizukubo and Hirashima 1987), and increased taxonomic relevance by uniting terminology between Mantodea and Blattodea. The revised terminologies established in Klass (1997) were analyzed cladistically in Klass and Meier (2006) and yielded a phylogenetic topology more congruent with molecular data (Inward et al. 2007) than systems closer to McKittrick (1964)’s (Grandcolas 1996). As such, we follow Anisyutkin (2016) in using the terminology of Klass (1997) with only minor changes consistent with both systems (Table 1).

**Table 1.** Blaberidae genitalia terminology used in this study and previous works.

Term used in present work	Term used by Roth^1^	Corresponding term in Klass, 1997^2^	Corresponding term in Anisyutkin, 2016^3^
L2’	L2d + *lve*	L2’	L2D
L10’	prepuce	L10’	membranous lobe of apical subsclerite L2D
*via*	L2d	L2E’ + L4N’/ *via*	L2D (apex)
*lve*	*lve*	L2D’/ *lve*	L2D
L3’	R2 (hooked sclerite)	L3’/ *hla*	L3
L4’	NA	L4’	L4
R1’ + R3’ + R4’ + R5’ or R’ phallomere	L1	R1’ + R2’ + R3’+ R4’ + R5’	R1 + R3 + R4 + R5

1. Roth (1969, 1970a, 1970d) largely followed the terminology of McKittrick (1964). However, “prepuce” is his term for the membrane surrounding “L2d” and the spines associated with this membrane. 2. In Klass (1997)’s terminology, the apostrophe character indicates that the homology is side-reversed. Hence, Klass (1997)’s L3’ is named L for “left” despite being physically present on the right side in Blaberidae and Pseudophyllodromiinae. See Klass (1997) for detailed descriptions of terminology and homologies. 3. Anisyutkin (2016) followed Klass (1997) with slight modifications, including omitting the apostrophe character to denote side-reversals (but Anisyutkin’s terminology is still homologically consistent with Klass 1997). Notably, Anisyutkin (2016) examined Epilamprinae, whereas Klass (1997) did not.

Terminology for spination of the anterior ventral margin of the fore-femur follows Roth (2003). We also use euplantulae (Roth and Willis 1952) as the preferred term for the pads on the tarsomere, although “pulvilli” is more commonly used in cockroach taxonomy (Roth 2003). Other abbreviations used for morphological features are given in Table 2.

**Table 2.** Common abbreviations of cockroach’s morphology used in this work.

Abbreviation	Description
SA plate	Supra-anal plate
SG plate	Sub-genital plate
AV margin	Anterior-ventral margin. Unless specified, this is used only to refer to the front femur.
s.b.	Setal brush on right phallomere.
T	Abdominal tergal segment specified by number (e.g., T1, T2, T7)

Regarding the morphological measurements, total body length was measured along the medial line from the most anterior point (head or pronotum) to the most posterior point of the abdomen. Tegmina and cerci length were measured from their attachment point to the most distal part of each structure. Pronotal length was measured along the medial line, and pronotal width was measured as the widest part of the pronotum.

All holotype specimens will be deposited in the Museo de Historia Natural – Universidad Nacional Mayor de San Marcos in Lima, Peru (**MUSM**). Paratype specimens are temporarily stored in the research collection of coauthor DAE, until they are deposited in MUSM and the Illinois Natural History Survey Insect Collection.

### Literature and database search

To compile the country-level and regional checklist, we did a literature search that relied primarily on geographic records reported in the Cockroach Species File (CSF) online database (Hopkins and Beccaloni 2024). Of the two geography-based papers to focus on cockroaches of Peru (Shelford 1912; Albuquerque 1964), only one had records of Blaberidae (Shelford 1912), which was added to the checklist. We quickly searched other large taxonomic compendia focusing on Neotropical collections (e.g., Rehn 1903, 1916, 1930; Hebard 1921, 1926), but found no records beyond what was reported on the CSF. We did not do an in-depth search to verify which of the country records were specific to the department of Madre De Dios.

We complemented these records with identified species on iNaturalist. We searched for “Blaberidae” and filtered by region for records from Madre De Dios, Peru. We examined every observation and added specific identifications based on our knowledge of the fauna gained from our firsthand taxonomic work (reported here), and knowledge of the literature. While there were numerous records of *Blaberus* and *Panchlora* identified to the species level, we did not include these in the checklist due to poor species definitions for these taxa (Roth 1969, 1971b), and the lack of genitalia for us to examine. For all iNaturalist observations made by the authors of this work, we simply report those as new observations in our checklist, and not iNaturalist observations. Finally, the above search was expanded to include a larger area of the Amazon immediately neighboring Madre De Dios in Brazil and Bolivia. Since there are no distinct biogeographical boundaries, we considered the few additional taxa appearing in this wider area probably occurring in Madre De Dios as well.

## Systematic entomology

**Blaberidae Saussure, 1864**

### Epilamprinae Brunner von Wattenwyl, 1865

**Systematic scope**. *Epilampra*, *Poeciloderrhis* Stål, 1874, *Parapoeciloderrhis* Anisyutkin, 2016, *Phoraspidini* Rehn, 1951, *Cariacasia*Rehn 1928, *Litopeltis* Hebard, 1928, Poroblattini Roth, 1971, *Pinaconota* Saussure, 1895, *Homalopteryx* Brunner von Wattenwyl, 1865, *Thanatophyllum* Grandcolas, 1991, *Capucinella* Hebard, 1920.

**Notes**. We are including *Antioquita* Hebard, 1933 within Poroblattini (based on comparison of *Antioquita punctigera* Hebard, 1933 with other Poroblattini in Roth (1971a)).

Epilamprinae, as traditionally conceived (e.g., Shelford 1910; hereafter, Epilamprinae*sensu lato*), is polyphyletic. This is widely supported by molecular phylogenetics analyses (Legendre et al. 2017; Liu et al. 2023; Wang et al. 2023; Evangelista et al. 2024). However, Neotropical Epilamprinae taxa tested with phylogenomic data are likely monophyletic (Evangelista et al. 2024). Consistent with this, we consider Epilamprinae*sensu stricto* to be the monophyletic group containing *Epilampra*, which has thus far been demonstrated to be purely Neotropical (although testing of further taxa may eventually prove this clade to be more wide-spread).

**Figure 1.**
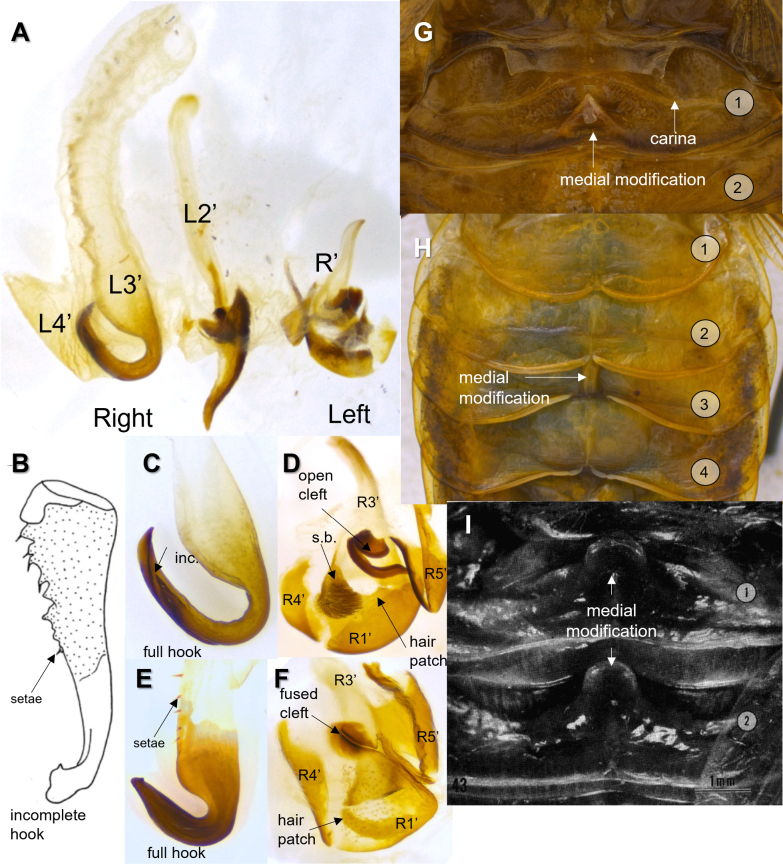
Overview of morphology utilized in the present work. **A**. Male genitalia from ventral view some sclerites labelled and side of body labelled; **B**. L3’ highlighting setae on the L3’ base/sheath, and the incomplete hook of L3’ (short and stout, turned distally but not strongly curved hook) contrasted with **C, E**. Fully curving hooks with and other details (subapical incision = inc. and setae); **D, F**. Showing details of R’ phallomere with cleft sclerite (fused and unfused), setal brush (s.b.), position of the hair patch, and possible homologies of sclerites (Anisyutkin 2016); **G–I**. Various tergal gland modification types, with tergal numbers labelled. Specimens imaged: (**A, C**) *Epilampra grisea* AUDE-PE-1-85, **B***Poeciloderrhis verticalis* (Burmeister, 1838) as illustrated in Anisyutkin (2016), (**D**) *E. conferta* UIRB-PE-26-48, (**E–F**) holotype of *E. sofia* sp. nov. UIRB-PE-26-37, (**G**) holotype of *E. homage* sp. nov. AUDE-PE-1-88, (**H**) holotype of *E. paititi* sp. nov. AUDE-PE-1-83, and (**I**) *Poeciloderrhis atriventris* (Saussure, 1895) as illustrated by Roth (1970a).

**Figure 2.**
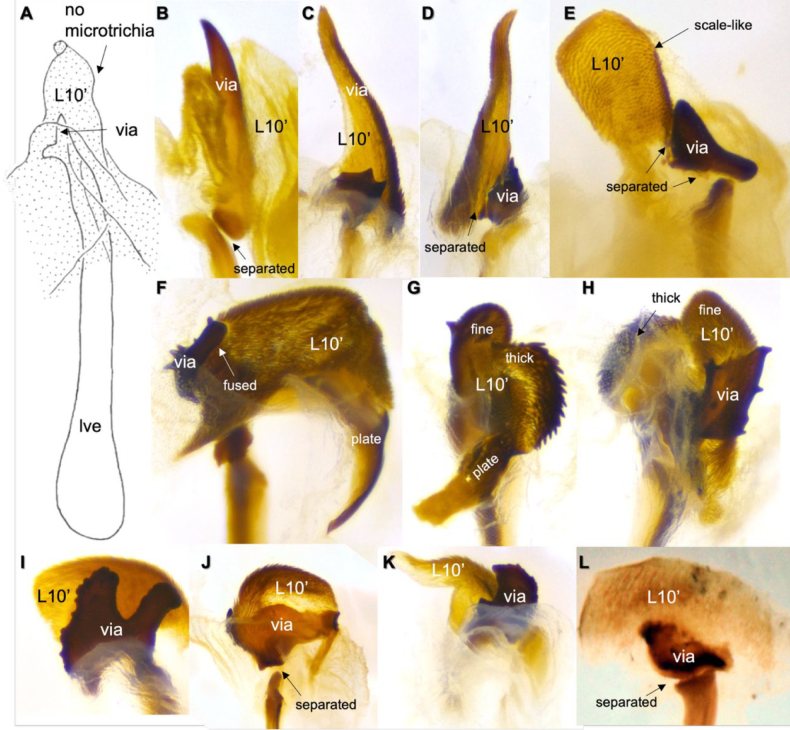
Overview of variation in male genital sclerite L2’ morphology. **A**. *lve* continuous with *via* is contrasted against (**B, D, E, J, L**) *via* being separated from *lve*. **A** L10’ (or the membrane typically bearing L10’) lacking any microtrichia is contrasted against; **B**. L10’ with lightly pigmented microtrichia; **C, D, I–L**. L10’ with densely pigmented microtrichia, (**F–H**) L10’ with two types of densely pigmented microtrichia, and **E**. Microtrichia modified into a scale-like surface. (**E**) *via* separated from L10’ is contrasted with **F–H**. *via* fused to L10’. **F, G**. L10’ with a plated sclerite and **G, H**. L10’ with a bar of thick microtrichia. Images of: (**A**) *Poeciloderrhis verticalis* as illustrated in Anisyutkin (2016), (**B**) holotype of *E. sofia* sp. nov. UIRB-PE-26-37, (**C–D**) *E. grisea* AUDE-PE-1-85, (**E**) E. sp. cf. azteca AUDE-PE-3-96, (**F–H**) holotype of *E. paititi* sp. nov. AUDE-PE-1-83, (**I**) holotype of *E. homage* sp. nov. AUDE-PE-1-88, (**J**) *E. conferta* UIRB-PE-26-48, (**K**) *E. yompori* sp. nov. holotype UIRB-PE-23-76, and (**L**) *Cariacasia capucina* Rehn, 1928 (https://cockroach.speciesfile.org/otus/857656).

**Remarks**. Much of the taxonomic work below, particularly among *Epilampra* species, involves considerations of morphological characters that disambiguate multiple Epilamprinae genera, some of which are not known from Peru. The new taxa we describe slightly change the distribution of diagnostic characters for these genera. As such, we provide a key with a visual guide (Figs 1, 2) to diagnose these genera (and species groups) from adult males. The key includes more Epilamprinae genera than are known from Peru. The data for this key are drawn primarily from the literature (Roth 1970a, 1970d, 1971a, 1972; Oliveira Cardoso da Silva and Lopes 2014; Lopes and Cardoso de Oliveira da Silva 2015; Anisyutkin 2016) with modifications based on our own findings. (NOTE: An interactive version of this key is available on https://cockroach.speciesfile.org).

### Dichotomous key to some Epilamprinae Brunner von Wattenwyl, 1865

**Table:** 

1	*via* clearly separated from *lve* (Fig. 2B, E, J, L) and forming a highly elongated, thin, and blunt flange. L10’ absent. Body usually cylindrical	**Poroblattini (including *Galiblatta* )**
–	Male genitalia without the combination above. Body typically cockroach-like (more-or-less flat) and not cylindrical	**2**
2	*via* clearly separated from *lve* (Fig. 2B, E, J, L) but forming a simple lump reaching only about 40% the length of the pigmented portion of L10’ (Fig. 2L) and L10’ forming a simple broad arch/rounded shape (Fig. 2L)	***Litopeltis* or *Cariacasia***
–	Male genitalia not with the combination above	**3**
3	Eyes bulging (anteriorly, outline of head noticeably raised at the compound eyes) or not bulging. If eyes not bulging, then phallomere L3’ short and stout, turned distally but not forming a strongly curved hook (i.e., incomplete hook; see Fig. 1B)	**4**
–	Eyes never bulging (anteriorly, outline of head straight/arched) and phallomere L3’ long and forming a full hook shape (Fig. 1C, E)	**6**
4	Eyes bulging and pronotum semi-circular or trapezoidal (mostly lacking the posterior-medial bulge typical of *Epilampra* and *Gyna*). No separation in L2’ and *via* absent or so highly reduced that it does not (or barely) protrudes from *lve* into the L10’ membrane. Adults tear-drop shaped from dorsal habitus, often with brilliant coloration	*** Phoraspis ***
–	Eyes bulging and pronotum semi-circular or trapezoidal (mostly lacking the posterior-medial bulge typical of *Epilampra* and *Gyna*). *via* separated from *lve*. Adults not significantly tear-drop shaped (i.e., distal ends of tegmina do not draw into a sharp point), and with pale (but mottled) coloration	***Homalopteryx* and *Thanatophyllum***
–	Not as in 4a or 4b (eyes not bulging, never tear-drop shaped, pronotum with a noticeable medio-posterior bulge). If *via* appears to be absent, the gestalt would be more like *Epilampra* and not tear-drop shaped or brilliantly colored	**5**
5	Terga 1 and 2 (T1 + T2) both highly modified (Fig. 1I), *via* forming a pointed shape solidly fused to *lve* (Fig. 2A), L10’ lacking microtrichia (Fig. 2A), and cleft of R’ phallomere fused together (Fig. 1F)	*** Poeciloderrhis ***
–	T1 and T2 without any modifications, *via* L-shaped and separated from *lve*, L10’ with dense microtrichia, and cleft of R’ phallomere not fused	***Epilampra carinulata* species group**
6	*via* forming a knob-like bulb distally and solidly connected to *lve*, T-shaped carina on T1 but otherwise no distinct tergal modifications (Anisyutkin 2016)	*** Parapoeciloderrhis ***
–	*via* not forming a bulb-like shape, *via* separated from *lve* (or lacking/fused and non-distinguishable), carina or tergal modification in T1 may or may not be present (but not T-shaped; Fig. 1G)	**7 (*Epilampra* except *carinulata* species group)**
7	R’ phallomere setal brush present (Fig. 1G)	**8**
–	R’ phallomere setal brush not present but a diffuse patch of pale hairs may or may not be present (Fig. 1F)	**10**
8	Tip of L3’ nipple-shaped and L10’ mostly localized to one side (right), and R’ phallomere setal brush extending behind the cleft (Roth 1970d)	***Epilampra yersiniana* species group**
–	Tip of L3’ not nipple-shaped, L10’ usually extending to both sides of *via*, and R’ phallomere setal brush present (but may be only lightly pigmented)	**9**
9	*via* formed into a flattened plate that is fused to L10’ (Fig. 2J)	***Epilampra mexicana* species group**
–	*via* thick (variably shaped) and laying above L10’ (Fig. 2 C, D)	***Epilampra abdomennigrum* species group**
10	*via* absent (or fused to *lve* and not-distinguishable)	***Epilampra shelfordi* species group**
–	*via* present	**11**
11	*via* shaped like a long claw, L10’ lacking microtrichia or with lightly pigmented microtrichia, T3 and/or T4 may or may not be modified (Figs 1H, 1F, 2B)	***Epilampra sodalis* species group**
–	*via* not claw-shaped, L10’ with microtrichia (or if lacking microtrichia, surface is scale-like in appearance), terga may or may not be modified	**12**
12	*via* fused to L10’ (Fig. 2F)	**13**
–	*via* separate and laying above L10’ (e.g., Fig. 2E)	*** Epilampra *** ^**1**^ ** burmeisteri species group**
13	L10’ with two types of microtrichia, fine and robust, and a plated sclerite (Fig. 2F–H). Tergal specializations present on T3 and T4 (Fig. 1H)	***Epilampra paititi* species group**
–	L10’ with a single type of microtrichia (fine) and no plated sclerite or tergal specializations	***Epilampra heusseriana* species group**

### Poroblattini Roth, 1971

***Galiblatta* Hebard, 1926**

#### *Galiblatta* sp.

Fig. 3, Table 3

**Figure 3.**
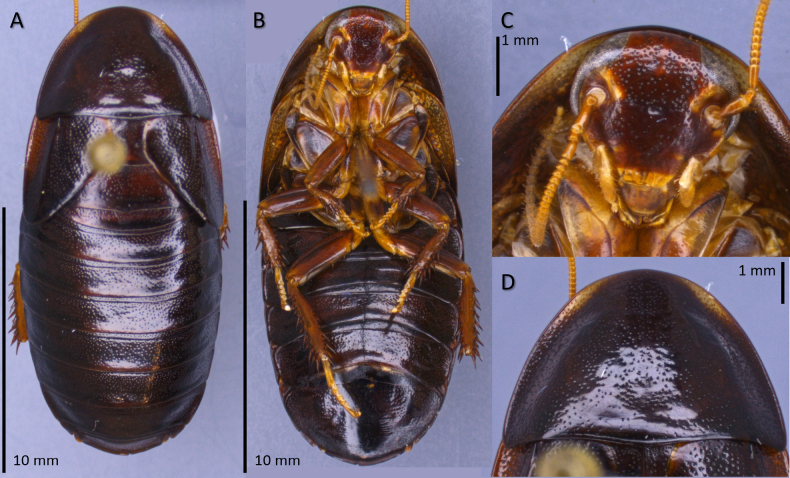
*Galiblatta* sp. adult female, UIRB-PE-20-70. **A**. Dorsal habitus; **B**. ventral habitus; **C**. details of head; **D**. pronotum. Scale bars: 10 mm (**A, B**); 1 mm (**C, D**).

**Table 3.** Morphological measurements of some Blaberidae species examined. F: female; M: male.

Taxon	Specimen	Sex	Measurement (mm)				
*Taxon*	*Specimen*	*Sex*	Total body length	Tegmina length	Pronotum length	Pronotum width	Cerci length
*Galiblatta* sp.	UIRB-PE-20-70	F	16.8	4.3	4.2	6.6	0.4
*Epilampra yompori* sp. nov.	UIRB-PE-23-76	M	26.9	25.6	6.4	8.1	3.6
*Epilampra yompori* sp. nov.	UIRB-PE-25-42	F	28	27.2	6.5	10	2.2
*Epilampra homage* sp. nov.	AUDE-PE-1-88	M	18.5 ^1^	17.9	4.5	6.1	2.1^2^
*Epilampra wandpero* sp. nov.	AUDE-PE-03-86	M	18.2	18.6	4.6	6.14	2.6^1^
Epilampra sp. cf. azteca	AUDE-PE-3-96	F	17.3	19.3	4.7	5.7	2.1
*Epilampra conferta “*placa*”*	AUDE-PE-1-84	M	23.5	25^1^	5.6	7.8	2.6
*Epilampra conferta “*placa*”*	AUDE-PE-1-87	F	33.8	30.5	7.4	9.8	2.8
*Epilampra conferta “*barra*”*	AUDE-PE-1-82	M	20.8	21.5	5.7	7.2	1.9
*Epilampra sofia* sp. nov.	UIRB-PE-26-37	M	23.3	22.6	5.5	6.3	2.33
*Epilampra sofia* sp. nov.	UIRB-PE-25-77	F	27.4	26.1	7.4	8.4	2.2
*Epilampra paititi* sp. nov.	AUDE-PE-1-83	M	22.5	25.6	5.2	6.7	2.5
*Lanxoblatta* sp. nov.	AUDE-PE-1-69	M	21.6	19.6	5.5	9.5	1.4
*Lanxoblatta* sp. nov.	UIRB-PE-26-74	F	21.3	11.8	6.3	11.5	1.2
*Phoetalia amigo* sp. nov.	AUDE-PE-1-95	M	13.6	12.4	3.4	4.9	0.9
*Phoetalia amigo* sp. nov.	UIRB-PE-30-77	F	19.1	14.2	4.2	6	1

1 The feature is broken and the measurement is estimated. 2 Taken from AUDE-PE-3-64.

**Material examined**. • 2 adult females: UIRB-PE-20-70, UIRB-PE-21-07. 5 juveniles: UIRB-PE-22-51, UIRB-PE-22-53, UIRB-PE-29-66, UIRB-PE-30-78, UIRB-PE-32-15 (locality and other data for all specimens are given in Table 4).

**Table 4.** Occurrence records for each Blaberidae specimen collected in Madre de Dios, Peru.

Catalog code	Number of individuals	Sex	Life Stage	Collection date	habitat	Sampling method	Collector	Country	Province	Locality	Altitude	Locality remarks	Latitude, Longitude	Type status	Scientific name
UIRB-PE-20-70	1	female	adult	2024-7-25		Hand collected	Emmy F. Medina E., Jared Martin, Johanna Schwartz, Dominic Evangelista	Peru	Madre de Dios	Kawsay Biological Station	193	Lindero trail or near station in concession	-12.537, -69.004		*Galiblatta* sp.
UIRB-PE-21-07	1	female	adult	2024-7-18	in wood with Passalidae	Hand collected	Emmy F. Medina E., Jared Martin, Johanna Schwartz, Dominic Evangelista	Peru	Madre de Dios	Kawsay Biological Station	175	Within concession, near station, atnight	-12.5282, -69.0141		*Galiblatta* sp.
UIRB-PE-22-51	1		juvenile	2024-7-18		Hand collected	Emmy F. Medina E., Jared Martin, Johanna Schwartz, Dominic Evangelista	Peru	Madre de Dios	Kawsay Biological Station	175	Within concession, near station, atnight	-12.5282, -69.0141		*Galiblatta* sp.
UIRB-PE-22-53	1		juvenile	2024-7-18		Hand collected	Emmy F. Medina E., Jared Martin, Johanna Schwartz, Dominic Evangelista	Peru	Madre de Dios	Kawsay Biological Station	175	Within concession, near station, atnight	-12.5282, -69.0141		* Galiblatta *
UIRB-PE-29-66	1		juvenile	2024-7-17 to 20		Hand collected	Emmy F. Medina E., Jared Martin, Johanna Schwartz, Dominic Evangelista	Peru	Madre de Dios	Kawsay Biological Station	175	Collected in or near camp	-12.5267, -69.0162		* Galiblatta *
UIRB-PE-30-78	1		juvenile	2024-7-25		Hand collected	Emmy F. Medina E., Jared Martin, Johanna Schwartz, Dominic Evangelista	Peru	Madre de Dios	Kawsay Biological Station	193	Lindero trail or near station in concession	-12.537, -69.004		* Galiblatta *
UIRB-PE-32-15	1		juvenile	2024-7-18		Hand collected	Emmy F. Medina E., Jared Martin, Johanna Schwartz, Dominic Evangelista	Peru	Madre de Dios	Kawsay Biological Station	175	Within concession, near station, atnight	-12.5282, -69.0141		* Galiblatta *
AUDE-PE-3-76	1		juvenile	2021-7-1			Evangelista-Huaman, Evangelista-Huaman, Medina Espinoza	Peru	Madre de Dios	Los Amigos Research Station		Cocha Lobos trail	-12.571056, -70.094889		*Epilampra yompori* Vanker, Medina-Espinoza, Evangelista
UIRB-PE-23-76	1	male	adult	2024-7-13		Hand collected	Emmy F. Medina E., Jared Martin, Johanna Schwartz, Dominic Evangelista	Peru	Madre de Dios	Finca Las Piedras Research Station	265	Trails in forest, near station at night	-12.2285, -69.1143	holotype of *Epilampra yompori*	*Epilampra yompori* Vanker, Medina-Espinoza, Evangelista
UIRB-PE-23-93	1		juvenile	2024-7-24		Hand collected	Emmy F. Medina E., Jared Martin, Johanna Schwartz, Dominic Evangelista	Peru	Madre de Dios	Kawsay Biological Station	185	Probably collected on trails D and X at night	-12.5364, -69.0084		*Epilampra yompori* Vanker, Medina-Espinoza, Evangelista
UIRB-PE-25-35	1		juvenile	2024-7-20		Hand collected	Emmy F. Medina E., Jared Martin, Johanna Schwartz, Dominic Evangelista	Peru	Madre de Dios	Kawsay Biological Station	175	Within concession, near station, atnight	-12.5282, -69.0141		*Epilampra yompori* Vanker, Medina-Espinoza, Evangelista
UIRB-PE-25-36	1		juvenile	2024-7-20		Hand collected	Emmy F. Medina E., Jared Martin, Johanna Schwartz, Dominic Evangelista	Peru	Madre de Dios	Kawsay Biological Station	175	Within concession, near station, atnight	-12.5282, -69.0141		*Epilampra yompori* Vanker, Medina-Espinoza, Evangelista
UIRB-PE-25-42	1	female	adult	2024-7-16		Hand collected	Emmy F. Medina E., Jared Martin, Johanna Schwartz, Dominic Evangelista	Peru	Madre de Dios	Finca Las Piedras Research Station	264	Night hike (Lindero, Tapir, Unguruhaui, Castana trails)	-12.2262, -69.1124		*Epilampra yompori* Vanker, Medina-Espinoza, Evangelista
UIRB-PE-25-78	1		juvenile	2024-7-15		Hand collected	Emmy F. Medina E., Jared Martin, Johanna Schwartz, Dominic Evangelista	Peru	Madre de Dios	Finca Las Piedras Research Station	264	Anaconda and Tapir trail, night hike	-12.2273, -69.1075		*Epilampra yompori* Vanker, Medina-Espinoza, Evangelista
UIRB-PE-26-23	1		juvenile	2024-7-23		Hand collected	Emmy F. Medina E., Jared Martin, Johanna Schwartz, Dominic Evangelista	Peru	Madre de Dios	Kawsay Biological Station	185	Trail E (atnight)	-12.536, -69.008		*Epilampra yompori* Vanker, Medina-Espinoza, Evangelista
UIRB-PE-26-25	1		juvenile	2024-7-23		Hand collected	Emmy F. Medina E., Jared Martin, Johanna Schwartz, Dominic Evangelista	Peru	Madre de Dios	Kawsay Biological Station	185	Trail E (atnight)	-12.536, -69.008		*Epilampra yompori* Vanker, Medina-Espinoza, Evangelista
UIRB-PE-26-38	1		juvenile	2024-7-11		Hand collected	Emmy F. Medina E., Jared Martin, Johanna Schwartz, Dominic Evangelista	Peru	Madre de Dios	Finca Las Piedras Research Station	265	Trails in forest near station at night	-12.2258, -69.1143		*Epilampra yompori* Vanker, Medina-Espinoza, Evangelista
UIRB-PE-31-51	1		juvenile	2024-7-15		Hand collected	Emmy F. Medina E., Jared Martin, Johanna Schwartz, Dominic Evangelista	Peru	Madre de Dios	Finca Las Piedras Research Station	264	Anaconda and Tapir trail, night hike	-12.2273, -69.1075		*Epilampra yompori* Vanker, Medina-Espinoza, Evangelista
AUDE-PE-1-85	1	male	adult	2021-6-25		night. Hand collected	Evangelista-Huaman, Evangelista-Huaman, Medina Espinoza	Peru	Madre de Dios	Puerto Maldonado		Kapievi Ecovillage			*Epilampra grisea* (De Geer, 1773)
AUDE-PE-3-91	1	female	adult	2021-6-25 to 30		night. Hand collected	Evangelista-Huaman, Evangelista-Huaman, Medina Espinoza	Peru	Madre de Dios	Puerto Maldonado		Kapievi Ecovillage			*Epilampra grisea* (De Geer, 1773)
UIRB-PE-24-15	1	female	adult	2024-7-23		Hand collected	Emmy F. Medina E., Jared Martin, Johanna Schwartz, Dominic Evangelista	Peru	Madre de Dios	Kawsay Biological Station	185	Trail E at night	-12.536, -69.008		*Epilampra grisea* (De Geer, 1773)
UIRB-PE-26-53	1	female	adult	2024-7-24		Hand collected	Emmy F. Medina E., Jared Martin, Johanna Schwartz, Dominic Evangelista	Peru	Madre de Dios	Kawsay Biological Station	185	Probably collected on trails D and X at night	-12.5364, -69.0084		*Epilampra grisea* (De Geer, 1773)
UIRB-PE-26-54	1	male	adult	2024-7-22 to 23		Pitfall traps baited with fish in ground	Emmy F. Medina E., Jared Martin, Johanna Schwartz, Dominic Evangelista	Peru	Madre de Dios	Kawsay Biological Station	185	Lindero trail	-12.5316, -69.0072		*Epilampra grisea* (De Geer, 1773)
UIRB-PE-26-45	1	male	adult	2024-7-15		Hand collected	Emmy F. Medina E., Jared Martin, Johanna Schwartz, Dominic Evangelista	Peru	Madre de Dios	Finca Las Piedras Research Station	264	Anaconda and Tapir trail, night hike	-12.2273, -69.1075		*Epilampra opaca* Walker, 1868
UIRB-PE-26-56	1	male	adult	2024-7-11		Hand collected	Emmy F. Medina E., Jared Martin, Johanna Schwartz, Dominic Evangelista	Peru	Madre de Dios	Finca Las Piedras Research Station	265	Trails in forest near station at night	-12.2258, -69.1143		*Epilampra opaca* Walker, 1868
AUDE-PE-1-88	1	male	adult	2021-7-6		day. Hand collected	Evangelista-Huaman, Evangelista-Huaman, Medina Espinoza	Peru	Madre de Dios	Los Amigos Research Station		Plataforma trail	-12.563056, -70.103333	holotype of *Epilampra homage*	*Epilampra homage* Vanker, Medina-Espinoza, Evangelista
AUDE-PE-3-63	1	male	adult	2021-7-4		night. Hand collected	Evangelista-Huaman, Evangelista-Huaman, Medina Espinoza	Peru	Madre de Dios	Los Amigos Research Station		Lowland forest	-12.571389, -70.094444		*Epilampra homage* Vanker, Medina-Espinoza, Evangelista
AUDE-PE-3-64	1	male	adult	2021-7-4		night. Hand collected	Evangelista-Huaman, Evangelista-Huaman, Medina Espinoza	Peru	Madre de Dios	Los Amigos Research Station		Lowland forest	-12.571389, -70.094444		*Epilampra homage* Vanker, Medina-Espinoza, Evangelista
UIRB-PE-24-14	1	male	adult	2024-7-14		Hand collected	Emmy F. Medina E., Jared Martin, Johanna Schwartz, Dominic Evangelista	Peru	Madre de Dios	Finca Las Piedras Research Station	265	Trails in forest, near station at night	-12.2285, -69.1143		*Epilampra homage* Vanker, Medina-Espinoza, Evangelista
UIRB-PE-26-70	1	male	adult	2024-7-16		Hand collected	Emmy F. Medina E., Jared Martin, Johanna Schwartz, Dominic Evangelista	Peru	Madre de Dios	Finca Las Piedras Research Station	264	Night hike (Lindero, Tapir, Unguruhaui, Castana trails)	-12.2262, -69.1124		*Epilampra homage* Vanker, Medina-Espinoza, Evangelista
AUDE-PE-3-86	1	male	adult	2021-6-29	on forest floor	night. Hand collected	Evangelista-Huaman, Evangelista-Huaman, Medina Espinoza	Peru	Madre de Dios	Ecoaventuras Amazonica			-12.505556, -69.173889	holotype of *Epilampra wandpero*	*Epilampra wandpero* Vanker, Medina-Espinoza, Evangelista
AUDE-PE-2-85	1	male	adult	2021-6-25		night. Hand collected	Evangelista-Huaman, Evangelista-Huaman, Medina Espinoza	Peru	Madre de Dios	Puerto Maldonado		Kapievi Ecovillage	-12.612083, -69.196111		*Epilampra wandpero* Vanker, Medina-Espinoza, Evangelista
AUDE-PE-3-70	1	male	adult	2021-7-4		night. Hand collected	Evangelista-Huaman, Evangelista-Huaman, Medina Espinoza	Peru	Madre de Dios	Los Amigos Research Station		Lowland forest	-12.571389, -70.094444		*Epilampra wandpero* Vanker, Medina-Espinoza, Evangelista
AUDE-PE-3-72	1	male	adult	2021-6-26		night. Hand collected	Evangelista-Huaman, Evangelista-Huaman, Medina Espinoza	Peru	Madre de Dios	Puerto Maldonado		“Camungo” trail	-12.628833, -69.189694		*Epilampra wandpero* Vanker, Medina-Espinoza, Evangelista
AUDE-PE-3-80	1	male	adult	2021-7-1		night. Hand collected	Evangelista-Huaman, Evangelista-Huaman, Medina Espinoza	Peru	Madre de Dios	Puerto Maldonado		“Camungo” trail	-12.628833, -69.189694		*Epilampra wandpero* Vanker, Medina-Espinoza, Evangelista
AUDE-PE-3-90	1	male	adult	2021-7-4		night. Hand collected	Evangelista-Huaman, Evangelista-Huaman, Medina Espinoza	Peru	Madre de Dios	Los Amigos Research Station		Lowland forest	-12.571389, -70.094444		*Epilampra wandpero* Vanker, Medina-Espinoza, Evangelista
UIRB-PE-23-86	1	male	adult	2024-7-19		Hand collected	Emmy F. Medina E., Jared Martin, Johanna Schwartz, Dominic Evangelista	Peru	Madre de Dios	Kawsay Biological Station	175	Within concession, near station, atnight	-12.5282, -69.0141		*Epilampra wandpero* Vanker, Medina-Espinoza, Evangelista
UIRB-PE-23-94	1	male	adult	2024-7-19		Hand collected	Emmy F. Medina E., Jared Martin, Johanna Schwartz, Dominic Evangelista	Peru	Madre de Dios	Kawsay Biological Station	175	Within concession, near station, atnight	-12.5282, -69.0141		*Epilampra wandpero* Vanker, Medina-Espinoza, Evangelista
UIRB-PE-23-95	1	male	adult	2024-7-19		Hand collected	Emmy F. Medina E., Jared Martin, Johanna Schwartz, Dominic Evangelista	Peru	Madre de Dios	Kawsay Biological Station	175	Within concession, near station, atnight	-12.5282, -69.0141		*Epilampra wandpero* Vanker, Medina-Espinoza, Evangelista
UIRB-PE-23-96	1	male	adult	2024-7-19		Hand collected	Emmy F. Medina E., Jared Martin, Johanna Schwartz, Dominic Evangelista	Peru	Madre de Dios	Kawsay Biological Station	175	Within concession, near station, atnight	-12.5282, -69.0141		*Epilampra wandpero* Vanker, Medina-Espinoza, Evangelista
UIRB-PE-23-98	1	male	adult	2024-7-19		Hand collected	Emmy F. Medina E., Jared Martin, Johanna Schwartz, Dominic Evangelista	Peru	Madre de Dios	Kawsay Biological Station	175	Within concession, near station, atnight	-12.5282, -69.0141		*Epilampra wandpero* Vanker, Medina-Espinoza, Evangelista
UIRB-PE-24-01	1	male	adult	2024-7-18		Hand collected	Emmy F. Medina E., Jared Martin, Johanna Schwartz, Dominic Evangelista	Peru	Madre de Dios	Kawsay Biological Station	175	Within concession, near station, atnight	-12.5282, -69.0141		*Epilampra wandpero* Vanker, Medina-Espinoza, Evangelista
UIRB-PE-26-42	1	male	adult	2024-7-23		Hand collected	Emmy F. Medina E., Jared Martin, Johanna Schwartz, Dominic Evangelista	Peru	Madre de Dios	Kawsay Biological Station	185	Trail E at night	-12.536, -69.008		*Epilampra wandpero* Vanker, Medina-Espinoza, Evangelista
UIRB-PE-26-50	1	male	adult	2024-7-22		Hand collected	Emmy F. Medina E., Jared Martin, Johanna Schwartz, Dominic Evangelista	Peru	Madre de Dios	Kawsay Biological Station	193	Within concession, near station, atnight	-12.537, -69.004		*Epilampra wandpero* Vanker, Medina-Espinoza, Evangelista
UIRB-PE-26-51	1	male	adult	2024-7-22		Hand collected	Emmy F. Medina E., Jared Martin, Johanna Schwartz, Dominic Evangelista	Peru	Madre de Dios	Kawsay Biological Station	193	Within concession, near station, atnight	-12.537, -69.004		*Epilampra wandpero* Vanker, Medina-Espinoza, Evangelista
UIRB-PE-26-55	1	male	adult	2024-7-24		Hand collected	Emmy F. Medina E., Jared Martin, Johanna Schwartz, Dominic Evangelista	Peru	Madre de Dios	Kawsay Biological Station	185	Probably collected on trails D and X at night	-12.5364, -69.0084		*Epilampra wandpero* Vanker, Medina-Espinoza, Evangelista
UIRB-PE-26-71	1	male	adult	2024-7-17			Emmy F. Medina E., Jared Martin, Johanna Schwartz, Dominic Evangelista	Peru	Madre de Dios	Kawsay Biological Station	175	Collected in or near camp	-12.5282, -69.0141		*Epilampra wandpero* Vanker, Medina-Espinoza, Evangelista
UIRB-PE-26-41	1	female	adult	2024-7-23		Hand collected	Emmy F. Medina E., Jared Martin, Johanna Schwartz, Dominic Evangelista	Peru	Madre de Dios	Kawsay Biological Station	185	Trail E at night	-12.536, -69.008		*Epilampra wandpero* Vanker, Medina-Espinoza, Evangelista
UIRB-PE-26-43	1	female	adult	2024-7-23		Hand collected	Emmy F. Medina E., Jared Martin, Johanna Schwartz, Dominic Evangelista	Peru	Madre de Dios	Kawsay Biological Station	185	Trail E at night	-12.536, -69.008		*Epilampra wandpero* Vanker, Medina-Espinoza, Evangelista
UIRB-PE-26-49	1	female	adult	2024-7-22		Hand collected	Emmy F. Medina E., Jared Martin, Johanna Schwartz, Dominic Evangelista	Peru	Madre de Dios	Kawsay Biological Station	193	Within concession, near station, atnight	-12.537, -69.004		*Epilampra wandpero* Vanker, Medina-Espinoza, Evangelista
UIRB-PE-26-52	1	female	adult	2024-7-24		Hand collected	Emmy F. Medina E., Jared Martin, Johanna Schwartz, Dominic Evangelista	Peru	Madre de Dios	Kawsay Biological Station	185	Probably collected on trails D and X at night	-12.5364, -69.0084		*Epilampra wandpero* Vanker, Medina-Espinoza, Evangelista
UIRB-PE-26-72	1	female	adult	2024-7-17			Emmy F. Medina E., Jared Martin, Johanna Schwartz, Dominic Evangelista	Peru	Madre de Dios	Kawsay Biological Station	175	Collected in or near camp	-12.5282, -69.0141		*Epilampra wandpero* Vanker, Medina-Espinoza, Evangelista
AUDE-PE-3-92	1	female	adult	2021-7-4		night. Hand collected	Evangelista-Huaman, Evangelista-Huaman, Medina Espinoza	Peru	Madre de Dios	Los Amigos Research Station		Lowland forest	-12.571389, -70.094444		*Epilampra cf. azteca*
AUDE-PE-3-96	1	male	adult	2021-7-3		day. Hand collected	Evangelista-Huaman, Evangelista-Huaman, Medina Espinoza	Peru	Madre de Dios	Los Amigos Research Station		Plataforma trail	-12.563056, -70.103333		*Epilampra cf. azteca*
AUDE-PE-1-67	1	male	adult	2021-7-6		night. Hand collected	Evangelista-Huaman, Evangelista-Huaman, Medina Espinoza	Peru	Madre de Dios	Los Amigos Research Station		Trail 10	-12.566389, -70.099722		*Epilampra conferta* “barra”
AUDE-PE-1-82	1	male	adult	2021-7-6		night. Hand collected	Evangelista-Huaman, Evangelista-Huaman, Medina Espinoza	Peru	Madre de Dios	Los Amigos Research Station		Trail 10	-12.566389, -70.099722		*Epilampra conferta* “barra”
AUDE-PE-2-61	1	male	adult	2021-7-3		night. Hand collected	Evangelista-Huaman, Evangelista-Huaman, Medina Espinoza	Peru	Madre de Dios	Los Amigos Research Station		Plataforma trail	-12.565481, -70.1027		*Epilampra conferta* “barra”
AUDE-PE-2-63	1	male	adult	2021-7-3		day. Hand collected	Evangelista-Huaman, Evangelista-Huaman, Medina Espinoza	Peru	Madre de Dios	Los Amigos Research Station		Plataforma trail	-12.565481, -70.1027		*Epilampra conferta* “barra”
AUDE-PE-2-64	1	male	adult	2021-7-6		night. Hand collected	Evangelista-Huaman, Evangelista-Huaman, Medina Espinoza	Peru	Madre de Dios	Los Amigos Research Station		Trail 10	-12.566389, -70.099722		*Epilampra conferta* “barra”
AUDE-PE-2-66	1	male	adult	2021-7-6		night. Hand collected	Evangelista-Huaman, Evangelista-Huaman, Medina Espinoza	Peru	Madre de Dios	Los Amigos Research Station		Trail 10	-12.566389, -70.099722		*Epilampra conferta* “barra”
AUDE-PE-2-94	1	male	adult	2021-7-4		night. Hand collected	Evangelista-Huaman, Evangelista-Huaman, Medina Espinoza	Peru	Madre de Dios	Los Amigos Research Station		Lowland forest	-12.571389, -70.094444		*Epilampra conferta* “barra”
UIRB-PE-26-46	1	male	adult	2024-7-11		Hand collected	Emmy F. Medina E., Jared Martin, Johanna Schwartz, Dominic Evangelista	Peru	Madre de Dios	Finca Las Piedras Research Station		Trails in forest near station at night	-12.2258, -69.1143		*Epilampra conferta* “barra”
AUDE-PE-1-74	1	male	adult	2021-7-4		night. Hand collected	Evangelista-Huaman, Evangelista-Huaman, Medina Espinoza	Peru	Madre de Dios	Los Amigos Research Station		Lowland forest	-12.571389, -70.094444		*Epilampra conferta* “placa”
AUDE-PE-1-84	1	male	adult	2021-7-3		day. Hand collected	Evangelista-Huaman, Evangelista-Huaman, Medina Espinoza	Peru	Madre de Dios	Los Amigos Research Station		Plataforma trail	-12.565481, -70.1027		*Epilampra conferta* “placa”
AUDE-PE-1-86	1	male	adult	2021-7-4		night. Hand collected	Evangelista-Huaman, Evangelista-Huaman, Medina Espinoza	Peru	Madre de Dios	Los Amigos Research Station		Lowland forest	-12.571389, -70.094444		*Epilampra conferta* “placa”
AUDE-PE-1-87	1	female	adult	2021-7-4		night. Hand collected	Evangelista-Huaman, Evangelista-Huaman, Medina Espinoza	Peru	Madre de Dios	Los Amigos Research Station		Lowland forest	-12.571389, -70.094444		*Epilampra conferta* “placa”
AUDE-PE-3-59	1	male	adult	2021-6-29	on forest floor	night. Hand collected	Evangelista-Huaman, Evangelista-Huaman, Medina Espinoza	Peru	Madre de Dios	Ecoaventuras Amazonica			-12.505556, -69.173889		*Epilampra conferta* “placa”
AUDE-PE-3-68	1		adult	2021-7-4		night. Hand collected	Evangelista-Huaman, Evangelista-Huaman, Medina Espinoza	Peru	Madre de Dios	Los Amigos Research Station		Lowland forest	-12.571389, -70.094444		*Epilampra conferta* “placa”
AUDE-PE-3-69	1	male	adult	2021-7-3		night. Hand collected	Evangelista-Huaman, Evangelista-Huaman, Medina Espinoza	Peru	Madre de Dios	Los Amigos Research Station		Plataforma trail	-12.565481, -70.1027		*Epilampra conferta* “placa”
AUDE-PE-3-74	1	male	adult	2021-7-3		day. Hand collected	Evangelista-Huaman, Evangelista-Huaman, Medina Espinoza	Peru	Madre de Dios	Los Amigos Research Station		Plataforma trail	-12.565481, -70.1027		*Epilampra conferta* “placa”
AUDE-PE-3-75	1	male	adult	2021-7-6		night. Hand collected	Evangelista-Huaman, Evangelista-Huaman, Medina Espinoza	Peru	Madre de Dios	Los Amigos Research Station		Trail 17 & 2	-12.559556, -70.107667		*Epilampra conferta* “placa”
AUDE-PE-3-79	1	female	adult	2021-7-6		night. Hand collected	Evangelista-Huaman, Evangelista-Huaman, Medina Espinoza	Peru	Madre de Dios	Los Amigos Research Station		Trail 10	-12.566389, -70.099722		*Epilampra conferta* “placa”
AUDE-PE-3-87	1	male	adult	2021-7-4		night. Hand collected	Evangelista-Huaman, Evangelista-Huaman, Medina Espinoza	Peru	Madre de Dios	Los Amigos Research Station		Lowland forest	-12.571389, -70.094444		*Epilampra conferta* “placa”
UIRB-PE-23-77	1	female	adult	2024-7-24		Hand collected	Emmy F. Medina E., Jared Martin, Johanna Schwartz, Dominic Evangelista	Peru	Madre de Dios	Kawsay Biological Station		Probably collected on trails D and X at night	-12.5364, -69.0084		*Epilampra conferta* “placa”
UIRB-PE-23-80	1	female	adult	2024-7-21		Hand collected	Emmy F. Medina E., Jared Martin, Johanna Schwartz, Dominic Evangelista	Peru	Madre de Dios	Kawsay Biological Station		Lindero trail to concession edge (at night)	-12.532, -69.01		*Epilampra conferta* “placa”
UIRB-PE-23-97	1	male	adult	2024-7-11		Hand collected	Emmy F. Medina E., Jared Martin, Johanna Schwartz, Dominic Evangelista	Peru	Madre de Dios	Finca Las Piedras Research Station		Trails in forest, near station at night	-12.2285, -69.1143		*Epilampra conferta* “placa”
UIRB-PE-23-99	1	female	adult	2024-7-11		Hand collected	Emmy F. Medina E., Jared Martin, Johanna Schwartz, Dominic Evangelista	Peru	Madre de Dios	Finca Las Piedras Research Station		Trails in forest, near station at night	-12.2285, -69.1143		*Epilampra conferta* “placa”
UIRB-PE-24-02	1	male	adult	2024-7-18		Hand collected	Emmy F. Medina E., Jared Martin, Johanna Schwartz, Dominic Evangelista	Peru	Madre de Dios	Kawsay Biological Station		Within concession, near station, atnight	-12.5282, -69.0141		*Epilampra conferta* “placa”
UIRB-PE-24-03	1	male	adult	2024-7-18		Hand collected	Emmy F. Medina E., Jared Martin, Johanna Schwartz, Dominic Evangelista	Peru	Madre de Dios	Kawsay Biological Station		Within concession, near station, atnight	-12.5282, -69.0141		*Epilampra conferta* “placa”
UIRB-PE-24-04	1	male	adult	2024-7-13		Hand collected	Emmy F. Medina E., Jared Martin, Johanna Schwartz, Dominic Evangelista	Peru	Madre de Dios	Finca Las Piedras Research Station		Trails in forest, near station at night	-12.2285, -69.1143		*Epilampra conferta* “placa”
UIRB-PE-24-05	1	male	adult	2024-7-13		Hand collected	Emmy F. Medina E., Jared Martin, Johanna Schwartz, Dominic Evangelista	Peru	Madre de Dios	Finca Las Piedras Research Station		Trails in forest, near station at night	-12.2285, -69.1143		*Epilampra conferta* “placa”
UIRB-PE-24-06	1	male	adult	2024-7-13		Hand collected	Emmy F. Medina E., Jared Martin, Johanna Schwartz, Dominic Evangelista	Peru	Madre de Dios	Finca Las Piedras Research Station		Trails in forest, near station at night	-12.2285, -69.1143		*Epilampra conferta* “placa”
UIRB-PE-24-07	1	female	adult	2024-7-25		Hand collected	Emmy F. Medina E., Jared Martin, Johanna Schwartz, Dominic Evangelista	Peru	Madre de Dios	Kawsay Biological Station		Lindero trail or near station in concession	-12.537, -69.004		*Epilampra conferta* “placa”
UIRB-PE-24-09	1	female	adult	2024-7-23		Hand collected	Emmy F. Medina E., Jared Martin, Johanna Schwartz, Dominic Evangelista	Peru	Madre de Dios	Kawsay Biological Station		Trail E at night	-12.536, -69.008		*Epilampra conferta* “placa”
UIRB-PE-24-11	1	female	adult	2024-7-25		Hand collected	Emmy F. Medina E., Jared Martin, Johanna Schwartz, Dominic Evangelista	Peru	Madre de Dios	Kawsay Biological Station		Lindero trail or near station in concession	-12.537, -69.004		*Epilampra conferta* “placa”
UIRB-PE-26-44	1	male	adult	2024-7-15		Hand collected	Emmy F. Medina E., Jared Martin, Johanna Schwartz, Dominic Evangelista	Peru	Madre de Dios	Finca Las Piedras Research Station		Anaconda and Tapir trail, night hike	-12.2273, -69.1075		*Epilampra conferta* “placa”
UIRB-PE-26-48	1	male	adult	2024-7-14		Hand collected	Emmy F. Medina E., Jared Martin, Johanna Schwartz, Dominic Evangelista	Peru	Madre de Dios	Finca Las Piedras Research Station		Trails in forest, near station at night	-12.2285, -69.1143		*Epilampra conferta* “placa”
UIRB-PE-26-80	1	male	adult	2024-7-16			Emmy F. Medina E., Jared Martin, Johanna Schwartz, Dominic Evangelista	Peru	Madre de Dios	Finca Las Piedras Research Station		Night hike (Lindero, Tapir, Unguruhaui, Castana trails)	-12.2262, -69.1124		*Epilampra conferta* “placa”
UIRB-PE-25-77	1	female	adult	2024-7-18		Hand collected	Emmy F. Medina E., Jared Martin, Johanna Schwartz, Dominic Evangelista	Peru	Madre de Dios	Kawsay Biological Station	175	Within concession, near station, atnight	-12.5282, -69.0141		*Epilampra sofia* Vanker, Medina-Espinoza, Evangelista
UIRB-PE-26-37	1	male	adult	2024-7-18		Hand collected	Emmy F. Medina E., Jared Martin, Johanna Schwartz, Dominic Evangelista	Peru	Madre de Dios	Kawsay Biological Station	175	Within concession, near station, atnight	-12.5282, -69.0141	holotype of *Epilampra sofia*	*Epilampra sofia* Vanker, Medina-Espinoza, Evangelista
AUDE-PE-1-83	1	male	adult	2021-7-3		day. Hand collected	Evangelista-Huaman, Evangelista-Huaman, Medina Espinoza	Peru	Madre de Dios	Los Amigos Research Station		Plataforma trail	-12.565481, -70.1027	holotype of *Epilampra paititi*	*Epilampra paititi* Vanker, Medina-Espinoza, Evangelista
UIRB-PE-20-37	1		juvenile	2024-7-13		Hand collected	Emmy F. Medina E., Jared Martin, Johanna Schwartz, Dominic Evangelista	Peru	Madre de Dios	Finca Las Piedras Research Station	265	Trails in forest, near station at night	-12.2285, -69.1143		* Phortioeca *
UIRB-PE-20-38	1		juvenile	2024-7-13		Hand collected	Emmy F. Medina E., Jared Martin, Johanna Schwartz, Dominic Evangelista	Peru	Madre de Dios	Finca Las Piedras Research Station	265	Trails in forest, near station at night	-12.2285, -69.1143		* Phortioeca *
UIRB-PE-20-39	1		juvenile	2024-7-13		Hand collected	Emmy F. Medina E., Jared Martin, Johanna Schwartz, Dominic Evangelista	Peru	Madre de Dios	Finca Las Piedras Research Station	265	Trails in forest, near station at night	-12.2285, -69.1143		* Phortioeca *
UIRB-PE-20-40	1		juvenile	2024-7-13		Hand collected	Emmy F. Medina E., Jared Martin, Johanna Schwartz, Dominic Evangelista	Peru	Madre de Dios	Finca Las Piedras Research Station	265	Trails in forest, near station at night	-12.2285, -69.1143		* Phortioeca *
UIRB-PE-20-41	1		juvenile	2024-7-13		Hand collected	Emmy F. Medina E., Jared Martin, Johanna Schwartz, Dominic Evangelista	Peru	Madre de Dios	Finca Las Piedras Research Station	265	Trails in forest, near station at night	-12.2285, -69.1143		* Phortioeca *
UIRB-PE-20-42	1		juvenile	2024-7-13		Hand collected	Emmy F. Medina E., Jared Martin, Johanna Schwartz, Dominic Evangelista	Peru	Madre de Dios	Finca Las Piedras Research Station	265	Trails in forest, near station at night	-12.2285, -69.1143		* Phortioeca *
UIRB-PE-20-43	1		juvenile	2024-7-13		Hand collected	Emmy F. Medina E., Jared Martin, Johanna Schwartz, Dominic Evangelista	Peru	Madre de Dios	Finca Las Piedras Research Station	265	Trails in forest, near station at night	-12.2285, -69.1143		* Phortioeca *
UIRB-PE-20-44	1		juvenile	2024-7-13		Hand collected	Emmy F. Medina E., Jared Martin, Johanna Schwartz, Dominic Evangelista	Peru	Madre de Dios	Finca Las Piedras Research Station	265	Trails in forest, near station at night	-12.2285, -69.1143		* Phortioeca *
UIRB-PE-20-45	1		juvenile	2024-7-13		Hand collected	Emmy F. Medina E., Jared Martin, Johanna Schwartz, Dominic Evangelista	Peru	Madre de Dios	Finca Las Piedras Research Station	265	Trails in forest, near station at night	-12.2285, -69.1143		* Phortioeca *
UIRB-PE-26-59	1		juvenile	2024-7-16		Hand collected	Emmy F. Medina E., Jared Martin, Johanna Schwartz, Dominic Evangelista	Peru	Madre de Dios	Finca Las Piedras Research Station	264	Night hike (Lindero, Tapir, Unguruhaui, Castana trails)	-12.2262, -69.1124		* Phortioeca *
AUDE-PE-1-69	1	male	adult	2021-7-6		night. Hand collected	Evangelista-Huaman, Evangelista-Huaman, Medina Espinoza	Peru	Madre de Dios	Los Amigos Research Station		Trail 10	-12.566389, -70.099722	holotype of *Lanxoblatta patriciae*	*Lanxoblatta patriciae* Vanker, Medina-Espinoza, Evangelista
AUDE-PE-1-89	1	male	adult	2021-7-6		night. Hand collected	Evangelista-Huaman, Evangelista-Huaman, Medina Espinoza	Peru	Madre de Dios	Los Amigos Research Station		Trail 17 & 2	-12.559556, -70.107667		*Lanxoblatta patriciae* Vanker, Medina-Espinoza, Evangelista
AUDE-PE-1-90	1	male	adult	2021-7-2		night	Evangelista-Huaman, Evangelista-Huaman, Medina Espinoza	Peru	Madre de Dios	Los Amigos Research Station		in/ around buildings	-12.569139, -70.100361		*Lanxoblatta patriciae* Vanker, Medina-Espinoza, Evangelista
UIRB-PE-26-61	1	male	adult	2024-7-16		Hand collected	Emmy F. Medina E., Jared Martin, Johanna Schwartz, Dominic Evangelista	Peru	Madre de Dios	Finca Las Piedras Research Station		Night hike (Lindero, Tapir, Unguruhaui, Castana trails)	-12.2262, -69.1124		*Lanxoblatta patriciae* Vanker, Medina-Espinoza, Evangelista
UIRB-PE-26-62	1	female	adult	2024-7-16		Hand collected	Emmy F. Medina E., Jared Martin, Johanna Schwartz, Dominic Evangelista	Peru	Madre de Dios	Finca Las Piedras Research Station		Night hike (Lindero, Tapir, Unguruhaui, Castana trails)	-12.2262, -69.1124		*Lanxoblatta patriciae* Vanker, Medina-Espinoza, Evangelista
UIRB-PE-26-74	1	female	adult	2024-7-15		Hand collected	Emmy F. Medina E., Jared Martin, Johanna Schwartz, Dominic Evangelista	Peru	Madre de Dios	Finca Las Piedras Research Station		Anaconda and Tapir trail, night hike	-12.2273, -69.1075		*Lanxoblatta patriciae* Vanker, Medina-Espinoza, Evangelista
UIRB-PE-26-75	1	male	adult	2024-7-15		Hand collected	Emmy F. Medina E., Jared Martin, Johanna Schwartz, Dominic Evangelista	Peru	Madre de Dios	Finca Las Piedras Research Station		Anaconda and Tapir trail, night hike	-12.2273, -69.1075		*Lanxoblatta patriciae* Vanker, Medina-Espinoza, Evangelista
UIRB-PE-26-76	1	male	adult	2024-7-15	wood	Hand collected	Emmy F. Medina E., Jared Martin, Johanna Schwartz, Dominic Evangelista	Peru	Madre de Dios	Finca Las Piedras Research Station		Anaconda and Tapir trail, night hike	-12.2273, -69.1075		*Lanxoblatta patriciae* Vanker, Medina-Espinoza, Evangelista
UIRB-PE-26-77	1		juvenile	2024-7-15	wood	Hand collected	Emmy F. Medina E., Jared Martin, Johanna Schwartz, Dominic Evangelista	Peru	Madre de Dios	Finca Las Piedras Research Station		Anaconda and Tapir trail, night hike	-12.2273, -69.1075		*Lanxoblatta patriciae* Vanker, Medina-Espinoza, Evangelista
UIRB-PE-26-81	1		juvenile	2024-7-20	wood	Hand collected	Emmy F. Medina E., Jared Martin, Johanna Schwartz, Dominic Evangelista	Peru	Madre de Dios	Kawsay Biological Station		Within concession, near station, atnight	-12.5282, -69.0141		*Lanxoblatta patriciae* Vanker, Medina-Espinoza, Evangelista
UIRB-PE-26-82	1		juvenile	2024-7-20		Hand collected	Emmy F. Medina E., Jared Martin, Johanna Schwartz, Dominic Evangelista	Peru	Madre de Dios	Kawsay Biological Station		Within concession, near station, atnight	-12.5282, -69.0141		*Lanxoblatta patriciae* Vanker, Medina-Espinoza, Evangelista
UIRB-PE-26-83	1		juvenile	2024-7-20		Hand collected	Emmy F. Medina E., Jared Martin, Johanna Schwartz, Dominic Evangelista	Peru	Madre de Dios	Kawsay Biological Station		Within concession, near station, atnight	-12.5282, -69.0141		*Lanxoblatta patriciae* Vanker, Medina-Espinoza, Evangelista
AUDE-PE-1-93	1		adult	2021-7-6	On wood	night. Hand collected	Evangelista-Huaman, Evangelista-Huaman, Medina Espinoza	Peru	Madre de Dios	Los Amigos Research Station		Trail 17 & 2	-12.559556, -70.107667		* Blaberus *
AUDE-PE-1-94	1		juvenile	2021-6-29	On wood	night. Hand collected	Evangelista-Huaman, Evangelista-Huaman, Medina Espinoza	Peru	Madre de Dios	Ecoaventuras Amazonica			-12.505556, -69.173889		* Blaberus *
AUDE-PE-1-97	1		juvenile	2021-7-3		day. Hand collected	Evangelista-Huaman, Evangelista-Huaman, Medina Espinoza	Peru	Madre de Dios	Los Amigos Research Station		Plataforma trail	-12.565481, -70.1027		* Blaberus *
AUDE-PE-1-98	1		juvenile	2021-7-30		night. Hand collected	Evangelista-Huaman, Evangelista-Huaman, Medina Espinoza	Peru	Madre de Dios	Puerto Maldonado		“Camungo” trail	-12.628833, -69.189694		* Blaberus *
UIRB-PE-20-01	1		juvenile	2024-7-12		Hand collected	Emmy F. Medina E., Jared Martin, Johanna Schwartz, Dominic Evangelista	Peru	Madre de Dios	Finca Las Piedras Research Station		Trails in forest, near station at night	-12.2285, -69.1143		* Blaberus *
UIRB-PE-20-02	1		juvenile	2024-7-12		Hand collected	Emmy F. Medina E., Jared Martin, Johanna Schwartz, Dominic Evangelista	Peru	Madre de Dios	Finca Las Piedras Research Station		Trails in forest, near station at night	-12.2285, -69.1143		* Blaberus *
UIRB-PE-20-03	1		juvenile	2024-7-22		Hand collected	Emmy F. Medina E., Jared Martin, Johanna Schwartz, Dominic Evangelista	Peru	Madre de Dios	Kawsay Biological Station		Within concession forestatnight	-12.537, -69.004		* Blaberus *
UIRB-PE-20-04	1		juvenile	2024-7-22		Hand collected	Emmy F. Medina E., Jared Martin, Johanna Schwartz, Dominic Evangelista	Peru	Madre de Dios	Kawsay Biological Station		Within concession forestatnight	-12.537, -69.004		* Blaberus *
UIRB-PE-20-05	1		juvenile	2024-7-22		Hand collected	Emmy F. Medina E., Jared Martin, Johanna Schwartz, Dominic Evangelista	Peru	Madre de Dios	Kawsay Biological Station		Within concession forestatnight	-12.537, -69.004		* Blaberus *
UIRB-PE-20-06	1		juvenile	2024-7-22		Hand collected	Emmy F. Medina E., Jared Martin, Johanna Schwartz, Dominic Evangelista	Peru	Madre de Dios	Kawsay Biological Station		Within concession forestatnight	-12.537, -69.004		* Blaberus *
UIRB-PE-20-07	1		juvenile	2024-7-24		Hand collected	Emmy F. Medina E., Jared Martin, Johanna Schwartz, Dominic Evangelista	Peru	Madre de Dios	Kawsay Biological Station		Probably collected on trails D and X at night	-12.5364, -69.0084		* Blaberus *
UIRB-PE-20-08	1		juvenile	2024-7-20		Hand collected	Emmy F. Medina E., Jared Martin, Johanna Schwartz, Dominic Evangelista	Peru	Madre de Dios	Kawsay Biological Station		Within concession, near station, atnight	-12.5282, -69.0141		* Blaberus *
UIRB-PE-20-09	1		juvenile	2024-7-20		Hand collected	Emmy F. Medina E., Jared Martin, Johanna Schwartz, Dominic Evangelista	Peru	Madre de Dios	Kawsay Biological Station		Within concession, near station, atnight	-12.5282, -69.0141		* Blaberus *
UIRB-PE-20-10	1		juvenile	2024-7-16		Hand collected	Emmy F. Medina E., Jared Martin, Johanna Schwartz, Dominic Evangelista	Peru	Madre de Dios	Finca Las Piedras Research Station		Night hike (Lindero, Tapir, Unguruhaui, Castana trails)	-12.2262, -69.1124		* Blaberus *
UIRB-PE-20-12	1		juvenile	2024-7-19		Hand collected	Emmy F. Medina E., Jared Martin, Johanna Schwartz, Dominic Evangelista	Peru	Madre de Dios	Kawsay Biological Station		Within concession, near station, atnight	-12.5282, -69.0141		* Blaberus *
UIRB-PE-20-13	1		juvenile	2024-7-19		Hand collected	Emmy F. Medina E., Jared Martin, Johanna Schwartz, Dominic Evangelista	Peru	Madre de Dios	Kawsay Biological Station		Within concession, near station, atnight	-12.5282, -69.0141		* Blaberus *
UIRB-PE-20-14	1		juvenile	2024-7-19		Hand collected	Emmy F. Medina E., Jared Martin, Johanna Schwartz, Dominic Evangelista	Peru	Madre de Dios	Kawsay Biological Station		Within concession, near station, atnight	-12.5282, -69.0141		* Blaberus *
UIRB-PE-20-15	1		juvenile	2024-7-19		Hand collected	Emmy F. Medina E., Jared Martin, Johanna Schwartz, Dominic Evangelista	Peru	Madre de Dios	Kawsay Biological Station		Within concession, near station, atnight	-12.5282, -69.0141		* Blaberus *
UIRB-PE-20-16	1		juvenile	2024-7-19		Hand collected	Emmy F. Medina E., Jared Martin, Johanna Schwartz, Dominic Evangelista	Peru	Madre de Dios	Kawsay Biological Station		Within concession, near station, atnight	-12.5282, -69.0141		* Blaberus *
UIRB-PE-20-17	1		juvenile	2024-7-19		Hand collected	Emmy F. Medina E., Jared Martin, Johanna Schwartz, Dominic Evangelista	Peru	Madre de Dios	Kawsay Biological Station		Within concession, near station, atnight	-12.5282, -69.0141		* Blaberus *
UIRB-PE-20-18	1		juvenile	2024-7-19	In wood with Passalidae	Hand collected	Emmy F. Medina E., Jared Martin, Johanna Schwartz, Dominic Evangelista	Peru	Madre de Dios	Kawsay Biological Station		Within concession, near station, atnight	-12.5282, -69.0141		* Blaberus *
UIRB-PE-20-21	1		juvenile	2024-7-19	In wood with Passalidae	Hand collected	Emmy F. Medina E., Jared Martin, Johanna Schwartz, Dominic Evangelista	Peru	Madre de Dios	Kawsay Biological Station		Within concession, near station, atnight	-12.5282, -69.0141		* Blaberus *
UIRB-PE-20-22	1		juvenile	2024-7-19		Hand collected	Emmy F. Medina E., Jared Martin, Johanna Schwartz, Dominic Evangelista	Peru	Madre de Dios	Kawsay Biological Station		Within concession, near station, atnight	-12.5282, -69.0141		* Blaberus *
UIRB-PE-20-23	1		juvenile	2024-7-19		Hand collected	Emmy F. Medina E., Jared Martin, Johanna Schwartz, Dominic Evangelista	Peru	Madre de Dios	Kawsay Biological Station		Within concession, near station, atnight	-12.5282, -69.0141		* Blaberus *
UIRB-PE-20-24	1		juvenile	2024-7-19		Hand collected	Emmy F. Medina E., Jared Martin, Johanna Schwartz, Dominic Evangelista	Peru	Madre de Dios	Kawsay Biological Station		Within concession, near station, atnight	-12.5282, -69.0141		* Blaberus *
UIRB-PE-20-25	1		juvenile	2024-7-19		Hand collected	Emmy F. Medina E., Jared Martin, Johanna Schwartz, Dominic Evangelista	Peru	Madre de Dios	Kawsay Biological Station		Within concession, near station, atnight	-12.5282, -69.0141		* Blaberus *
UIRB-PE-20-26	1		juvenile	2024-7-19		Hand collected	Emmy F. Medina E., Jared Martin, Johanna Schwartz, Dominic Evangelista	Peru	Madre de Dios	Kawsay Biological Station		Within concession, near station, atnight	-12.5282, -69.0141		* Blaberus *
UIRB-PE-20-27	1		juvenile	2024-7-24		Hand collected	Emmy F. Medina E., Jared Martin, Johanna Schwartz, Dominic Evangelista	Peru	Madre de Dios	Kawsay Biological Station		Probably collected on trails D and X at night	-12.5364, -69.0084		* Blaberus *
UIRB-PE-20-28	1		juvenile	2024-7-23		Hand collected	Emmy F. Medina E., Jared Martin, Johanna Schwartz, Dominic Evangelista	Peru	Madre de Dios	Kawsay Biological Station		Trail E at night	-12.536, -69.008		* Blaberus *
UIRB-PE-20-29	1		juvenile	2024-7-23		Hand collected	Emmy F. Medina E., Jared Martin, Johanna Schwartz, Dominic Evangelista	Peru	Madre de Dios	Kawsay Biological Station		Trail E at night	-12.536, -69.008		* Blaberus *
UIRB-PE-20-30	1		juvenile	2024-7-23		Hand collected	Emmy F. Medina E., Jared Martin, Johanna Schwartz, Dominic Evangelista	Peru	Madre de Dios	Kawsay Biological Station		Trail E at night	-12.536, -69.008		* Blaberus *
UIRB-PE-20-31	1		juvenile	2024-7-23		Hand collected	Emmy F. Medina E., Jared Martin, Johanna Schwartz, Dominic Evangelista	Peru	Madre de Dios	Kawsay Biological Station		Trail E at night	-12.536, -69.008		* Blaberus *
UIRB-PE-20-32	1		juvenile	2024-7-13		Hand collected	Emmy F. Medina E., Jared Martin, Johanna Schwartz, Dominic Evangelista	Peru	Madre de Dios	Finca Las Piedras Research Station		Trails in forest, near station at night	-12.2285, -69.1143		* Blaberus *
UIRB-PE-20-34	1		juvenile	2024-7-18		Hand collected	Emmy F. Medina E., Jared Martin, Johanna Schwartz, Dominic Evangelista	Peru	Madre de Dios	Kawsay Biological Station		Within concession, near station, atnight	-12.5282, -69.0141		* Blaberus *
UIRB-PE-20-36	1		juvenile	2024-7-18		Hand collected	Emmy F. Medina E., Jared Martin, Johanna Schwartz, Dominic Evangelista	Peru	Madre de Dios	Kawsay Biological Station		Within concession, near station, atnight	-12.5282, -69.0141		* Blaberus *
UIRB-PE-20-46	1		adult	2024-7-19		Hand collected	Emmy F. Medina E., Jared Martin, Johanna Schwartz, Dominic Evangelista	Peru	Madre de Dios	Kawsay Biological Station		At camp	-12.5282, -69.0141		* Blaberus *
UIRB-PE-20-47	1		adult	2024-7-25		Hand collected	Emmy F. Medina E., Jared Martin, Johanna Schwartz, Dominic Evangelista	Peru	Madre de Dios	Kawsay Biological Station		Lindero trail or near station in concession	-12.537, -69.004		* Blaberus *
UIRB-PE-20-48	1		adult	2024-7-25		Hand collected	Emmy F. Medina E., Jared Martin, Johanna Schwartz, Dominic Evangelista	Peru	Madre de Dios	Kawsay Biological Station		Lindero trail or near station in concession	-12.537, -69.004		* Blaberus *
UIRB-PE-20-69	1		juvenile	2024-7-18		Hand collected	Emmy F. Medina E., Jared Martin, Johanna Schwartz, Dominic Evangelista	Peru	Madre de Dios	Kawsay Biological Station		Within concession, near station, atnight	-12.5282, -69.0141		Hormeticini
UIRB-PE-21-04	1		juvenile	2024-7-22		Hand collected	Emmy F. Medina E., Jared Martin, Johanna Schwartz, Dominic Evangelista	Peru	Madre de Dios	Kawsay Biological Station		Within concession forestatnight	-12.537, -69.004		Hormeticini
UIRB-PE-21-20	1		juvenile	2024-7			Emmy F. Medina E., Jared Martin, Johanna Schwartz, Dominic Evangelista	Peru	Madre de Dios	Madre de Dios		Unk. Locality			Hormeticini
UIRB-PE-29-40	1		juvenile	2024-7-23		Hand collected	Emmy F. Medina E., Jared Martin, Johanna Schwartz, Dominic Evangelista	Peru	Madre de Dios	Kawsay Biological Station		Trail E at night	-12.536, -69.008		Hormeticini
UIRB-PE-30-76	1	male	adult	2024-7-20 to 23			Emmy F. Medina E., Jared Martin, Johanna Schwartz, Dominic Evangelista	Peru	Madre de Dios	Kawsay Biological Station			-12.5282, -69.0141		*Phoetalia pallida* (Brunner von Wattenwyl, 1865)
UIRB-PE-30-77	1	female	adult	2024-7-17 to 20			Emmy F. Medina E., Jared Martin, Johanna Schwartz, Dominic Evangelista	Peru	Madre de Dios	Kawsay Biological Station		Collected in or near camp	-12.5282, -69.0141		*Phoetalia amigo* Vanker, Medina-Espinoza, Evangelista
AUDE-PE-1-95	1	male	adult	2021-7-5 to 8		night.	Evangelista-Huaman, Evangelista-Huaman, Medina Espinoza	Peru	Madre de Dios	Los Amigos Research Station		in/ around buildings	-12.569139, -70.100361	holotype of *Phoetalia amigo*	*Phoetalia amigo* Vanker, Medina-Espinoza, Evangelista
AUDE-PE-1-70	1	male	adult	2021-7-3		day. Hand collected	Evangelista-Huaman, Evangelista-Huaman, Medina Espinoza	Peru	Madre de Dios	Los Amigos Research Station		Plataforma trail	-12.565481, -70.1027		*Hyporhicnoda humilior* Hebard, 1933
AUDE-PE-1-91	1		juvenile	2021-7-3 to 4		pitfall traps (bailed with beer)	Evangelista-Huaman, Evangelista-Huaman, Medina Espinoza	Peru	Madre de Dios	Los Amigos Research Station		Perro trail	-12.563028, -70.103556		*Hyporhicnoda humilior* Hebard, 1934
AUDE-PE-1-92	1		juvenile	2021-7-3		night. Hand collected	Evangelista-Huaman, Evangelista-Huaman, Medina Espinoza	Peru	Madre de Dios	Los Amigos Research Station		Plataforma trail	-12.565481, -70.1027		*Hyporhicnoda humilior* Hebard, 1935
AUDE-PE-2-92	1	male	adult	2021-7-6		LED white light trap at night in field	Evangelista-Huaman, Evangelista-Huaman, Medina Espinoza	Peru	Madre de Dios	Los Amigos Research Station			-12.569139, -70.100361		*Achroblatta luteola* (Blanchard, 1843)
AUDE-PE-2-97	1	male	adult	2021-7-6			Evangelista-Huaman, Evangelista-Huaman, Medina Espinoza	Peru	Madre de Dios	Los Amigos Research Station		in/ around buildings	-12.569139, -70.100361		*Achroblatta luteola* (Blanchard, 1843)
AUDE-PE-2-99	1	male	adult	2021-7-6		LED white light trap at night in field	Evangelista-Huaman, Evangelista-Huaman, Medina Espinoza	Peru	Madre de Dios	Los Amigos Research Station			-12.569139, -70.100361		*Achroblatta luteola* (Blanchard, 1843)
AUDE-PE-3-60	1	male	adult	2021-7-6		LED white light trap at night in field	Evangelista-Huaman, Evangelista-Huaman, Medina Espinoza	Peru	Madre de Dios	Los Amigos Research Station		in/ around buildings	-12.569139, -70.100361		* Panchlora *
AUDE-PE-3-62	1	male	adult	2021-7-6		LED white light trap at night in field	Evangelista-Huaman, Evangelista-Huaman, Medina Espinoza	Peru	Madre de Dios	Los Amigos Research Station		in/ around buildings	-12.569139, -70.100361		* Panchlora *
AUDE-PE-3-66	1	male	adult	2021-7-5		LED white light trap at night in field	Evangelista-Huaman, Evangelista-Huaman, Medina Espinoza	Peru	Madre de Dios	Los Amigos Research Station		in/ around buildings	-12.569139, -70.100361		* Panchlora *
AUDE-PE-3-67	1	male	adult	2021-7-6		LED white light trap at night in field	Evangelista-Huaman, Evangelista-Huaman, Medina Espinoza	Peru	Madre de Dios	Los Amigos Research Station		in/ around buildings	-12.569139, -70.100361		* Panchlora *
AUDE-PE-3-71	1	male	adult	2021-7-6		LED white light trap at night in field	Evangelista-Huaman, Evangelista-Huaman, Medina Espinoza	Peru	Madre de Dios	Los Amigos Research Station		in/ around buildings	-12.569139, -70.100361		* Panchlora *
AUDE-PE-3-73	1	male	adult	2021-7-6		LED white light trap at night in field	Evangelista-Huaman, Evangelista-Huaman, Medina Espinoza	Peru	Madre de Dios	Los Amigos Research Station		in/ around buildings	-12.569139, -70.100361		* Panchlora *
AUDE-PE-3-82	1	male	adult	2021-7-6		LED white light trap at night in field	Evangelista-Huaman, Evangelista-Huaman, Medina Espinoza	Peru	Madre de Dios	Los Amigos Research Station		in/ around buildings	-12.569139, -70.100361		* Panchlora *
AUDE-PE-3-83	1	male	adult	2021-7-5		LED white light trap at night in field	Evangelista-Huaman, Evangelista-Huaman, Medina Espinoza	Peru	Madre de Dios	Los Amigos Research Station		in/ around buildings	-12.569139, -70.100361		* Panchlora *
AUDE-PE-3-84	1	male	adult	2021-7-6		LED white light trap at night in field	Evangelista-Huaman, Evangelista-Huaman, Medina Espinoza	Peru	Madre de Dios	Los Amigos Research Station		in/ around buildings	-12.569139, -70.100361		* Panchlora *
AUDE-PE-3-85	1	male	adult	2021-7-6		LED white light trap at night in field	Evangelista-Huaman, Evangelista-Huaman, Medina Espinoza	Peru	Madre de Dios	Los Amigos Research Station		in/ around buildings	-12.569139, -70.100361		* Panchlora *
AUDE-PE-3-88	1	male	adult	2021-7-6		LED white light trap at night in field	Evangelista-Huaman, Evangelista-Huaman, Medina Espinoza	Peru	Madre de Dios	Los Amigos Research Station		in/ around buildings	-12.569139, -70.100361		* Panchlora *
AUDE-PE-3-93	1	male	adult	2021-7-5		LED white light trap at night in field	Evangelista-Huaman, Evangelista-Huaman, Medina Espinoza	Peru	Madre de Dios	Los Amigos Research Station		in/ around buildings	-12.569139, -70.100361		* Panchlora *
AUDE-PE-3-95	1	male	adult	2021-7-6		LED white light trap at night in field	Evangelista-Huaman, Evangelista-Huaman, Medina Espinoza	Peru	Madre de Dios	Los Amigos Research Station		in/ around buildings	-12.569139, -70.100361		* Panchlora *
AUDE-PE-4-58	1	female	adult	2021-7-1		night. Hand collected	Evangelista-Huaman, Evangelista-Huaman, Medina Espinoza	Peru	Madre de Dios	Puerto Maldonado		“Camungo” trail	-12.628833, -69.189694		* Panchlora *
AUDE-PE-4-59	1	male	adult	2021-7-6		LED white light trap at night in field	Evangelista-Huaman, Evangelista-Huaman, Medina Espinoza	Peru	Madre de Dios	Los Amigos Research Station		in/ around buildings	-12.569139, -70.100361		* Panchlora *
AUDE-PE-4-60	1	male	adult	2021-7-6		LED white light trap at night in field	Evangelista-Huaman, Evangelista-Huaman, Medina Espinoza	Peru	Madre de Dios	Los Amigos Research Station		in/ around buildings	-12.569139, -70.100361		* Panchlora *
AUDE-PE-4-61	1	male	adult	2021-7-6		LED white light trap at night in field	Evangelista-Huaman, Evangelista-Huaman, Medina Espinoza	Peru	Madre de Dios	Los Amigos Research Station		in/ around buildings	-12.569139, -70.100361		* Panchlora *
AUDE-PE-4-62	1	male	adult	2021-7-6		night. Hand collected	Evangelista-Huaman, Evangelista-Huaman, Medina Espinoza	Peru	Madre de Dios	Los Amigos Research Station		Trail 17 & 2	-12.559556, -70.107667		* Panchlora *
AUDE-PE-4-63	1	male	adult	2021-7-6		LED white light trap at night in field	Evangelista-Huaman, Evangelista-Huaman, Medina Espinoza	Peru	Madre de Dios	Los Amigos Research Station		in/ around buildings	-12.569139, -70.100361		* Panchlora *
AUDE-PE-9-51	1	male	adult	2021-7-6		LED white light trap at night in field	Evangelista-Huaman, Evangelista-Huaman, Medina Espinoza	Peru	Madre de Dios	Los Amigos Research Station		in/ around buildings	-12.569139, -70.100361		* Panchlora *
AUDE-PE-9-52	1	male	adult	2021-7-6		LED white light trap at night in field	Evangelista-Huaman, Evangelista-Huaman, Medina Espinoza	Peru	Madre de Dios	Los Amigos Research Station		in/ around buildings	-12.569139, -70.100361		* Panchlora *
AUDE-PE-9-53	1	male	adult	2021-7-5		LED light trap at night in field	Evangelista-Huaman, Evangelista-Huaman, Medina Espinoza	Peru	Madre de Dios	Los Amigos Research Station		in/ around buildings	-12.569139, -70.100361		* Panchlora *
AUDE-PE-9-54	1	male	adult	2021-7-5		LED white light trap at night in field	Evangelista-Huaman, Evangelista-Huaman, Medina Espinoza	Peru	Madre de Dios	Los Amigos Research Station		in/ around buildings	-12.569139, -70.100361		* Panchlora *
AUDE-PE-9-55	1	male	adult	2021-7-6		LED white light trap at night in field	Evangelista-Huaman, Evangelista-Huaman, Medina Espinoza	Peru	Madre de Dios	Los Amigos Research Station		in/ around buildings	-12.569139, -70.100361		* Panchlora *
AUDE-PE-9-56	1		adult	2021-7-6		LED light trap at night in field	Evangelista-Huaman, Evangelista-Huaman, Medina Espinoza	Peru	Madre de Dios	Los Amigos Research Station		in/ around buildings	-12.569139, -70.100361		* Panchlora *
AUDE-PE-9-58	1	male	adult	2021-7-5		LED light trap at night in field	Evangelista-Huaman, Evangelista-Huaman, Medina Espinoza	Peru	Madre de Dios	Los Amigos Research Station		in/ around buildings	-12.569139, -70.100361		* Panchlora *
AUDE-PE-9-59	1	male	adult	2021-7-5		LED white light trap at night in field	Evangelista-Huaman, Evangelista-Huaman, Medina Espinoza	Peru	Madre de Dios	Los Amigos Research Station		in/ around buildings	-12.569139, -70.100361		* Panchlora *
AUDE-PE-9-60	1	female	adult	2021-7-6		LED light trap at night in field	Evangelista-Huaman, Evangelista-Huaman, Medina Espinoza	Peru	Madre de Dios	Los Amigos Research Station		in/ around buildings	-12.569139, -70.100361		* Panchlora *
UIRB-PE-33-63	1	male	adult	2024-7-18	In Wood with Passalidae and Isoptera	Hand collected	Emmy F. Medina E., Jared Martin, Johanna Schwartz, Dominic Evangelista	Peru	Madre de Dios	Kawsay Biological Station		Within concession, near station, atnight	-12.5282, -69.0141		* Panchlora *
AUDE-PE-1-71	1		juvenile	2021-7-6		night. Hand collected	Evangelista-Huaman, Evangelista-Huaman, Medina Espinoza	Peru	Madre de Dios	Los Amigos Research Station		Trail 17 & 2	-12.559556, -70.107667		Panchlorinae
AUDE-PE-2-95	1		juvenile	2021-7-6		night. Hand collected	Evangelista-Huaman, Evangelista-Huaman, Medina Espinoza	Peru	Madre de Dios	Los Amigos Research Station		Trail 17 & 2	-12.559556, -70.107667		Panchlorinae
UIRB-PE-25-99	1		juvenile	2024-7-11		Hand collected	Emmy F. Medina E., Jared Martin, Johanna Schwartz, Dominic Evangelista	Peru	Madre de Dios	Finca Las Piedras Research Station	265	Trails in forest, near station at night	-12.2285, -69.1143		Panchlorinae
UIRB-PE-31-07	1		juvenile	2024-7-16		Hand collected	Emmy F. Medina E., Jared Martin, Johanna Schwartz, Dominic Evangelista	Peru	Madre de Dios	Finca Las Piedras Research Station	264	Night hike (Lindero, Tapir, Unguruhaui, Castana trails)	-12.2262, -69.1124		Panchlorinae

**Determination**. The adult female specimen was identified as Poroblattini based on the body shape (cylindrical), tegmina form (brachypterous), and pronotal coloration (with two very shallow windows just lateral of center). Finally, it was identified as *Galiblatta* based only on the similar coloration and tegmina shape to other *Galiblatta* spp. (Heleodoro et al. 2024). Juveniles were associated with adults based on overall morphological similarity.

**Remarks**. This genus is known to have cryptic species (Heleodoro et al. 2024). Lacking adult males or widespread genetic data, we could not determine this taxon to the species level.

**Range**. This is the first record of any *Galiblatta* sp. west of Manaus, Brazil (Heleodoro et al. 2024), and the first record in Peru.

**Habitat**. Flooded alluvial forest.

#### *Epilampra* Burmeister, 1838

**Remarks**. *Epilampra* is one of the most species-rich Blaberidae genera, with 69 species described prior to the present work (Hopkins and Beccaloni 2024). Despite its relatively high diversity, only one new species has been described since 2000. The taxonomic literature for the genus was disconnected until Roth (1970a) moved numerous species to *Poeciloderrhis* Stål, 1874 and Roth (1970d) made a revision of 30 *Epilampra* species. Roth (1970d) treated the genitalia of the genus for the first time, and revised groupings proposed by Rehn and Hebard (1927). Roth (1970d) also moved numerous *Audreia* spp. into the genus, a job that was later completed by Fisk and Schal (1981), who erected the novel *Carinulata* species group for the type species of *Audreia*. Anisyutkin (2016) recently described a similar genus, *Parapoeciloderrhis*, differentiated it from *Epilampra* and *Poeciloderrhis*, and made further descriptions of two *Epilampra* subgroups.

Roth (1970d) split the genus into “groups” and “subgroups”. Other authors followed his precedent and erected further groups (Fisk and Schal 1981). We maintain Roth’s terminology of “species groups” here. When integrating these classifications in https://cockroach.speciesfile.org the interface requires that we add them as superspecies.

Rehn and Hebard (1927) noted difficulties resulting from taxon descriptions based on superficial characteristics – an issue also discussed by later workers (Fisk and Schal 1981). Many of the species discussed below have variable color patterning on the pronotum, abdomen, and elsewhere, as well as variable intragender body size and allometry despite being from a single region of Peru (i.e., potentially a single population or metapopulation for each species). This highlights the danger in utilizing non-genital features for determining, diagnosing, or describing *Epilampra* taxa.

In justifying his placement of numerous *Epilampra* species in *Poeciloderrhis*, Roth (1970a) said: “the species of *Epilampra* in which males have tergal glands have markedly different genitalia than the species which lack these modifications.” Roth (1970d) would quickly contradict this, as he noted the presence of tergal glands in some undescribed *Epilampra* spp. Yet, these tergal modifications were notably different from *Poeciloderrhis*. Anisyutkin (2016) described *Parapoeciloderrhis*, which has a T-shaped carina on T1, but is otherwise lacking notable external tergal modifications. We describe multiple new species of *Epilampra* below, which have all these features (or very similar ones), but we have kept their placement in *Epilampra*. *Poeciloderrhis* still has numerous consistent differences from *Epilampra* and *Parapoeciloderrhis*, but the presence of a visible tergal gland (on T1) is no longer a reliable character. As such, we provide an updated diagnosis to males of the genus.

**Diagnosis of *Epilampra* males** (modified from Roth 1970d). Medial tergal modifications on both T1 and T2 not present (although modifications can be present on T1 alone, or other tergal segments; modifications on both T1 and T2 are present in *Poeciloderrhis*), *via* and *lve* separated from each other (vs fused), R’ phallomere with or without a setal brush (*Poeciloderrhis* and *Parapoeciloderrhis* lack a setal brush), and R’ phallomere cleft ancestrally not fused together (vs fused in *Poeciloderrhis*; except in the *Epilampra sodalis* group, which may have fully or partially fused R’ phallomere cleft). Males usually without any tergal modifications on tergal segments 1 or 2. *via* usually not like a simple pointed spear-tip (like a simple pointed spear-tip in *Poeciloderrhis*) or curved and flattened into a disk apically (as in *Parapoeciloderrhis*). L10’ usually distinctly outlined and setose (L10’ highly reduced in *Poeciloderrhis* and *Parapoeciloderrhis*). Distal portion of L3’ with a full-hook shape (Fig. 1C, E; except in the *carinulata* species group), and with a subapical incision (except in the *carinulata*, *shelfordi*, and *yersiniana* species groups).

##### Epilampra yompori

Taxon classificationAnimaliaBlattodeaBlaberidae

Vanker, Medina-Espinoza, & Evangelista sp. nov.

4D1BE96F-215D-5931-8480-81E6B9A068AF

https://zoobank.org/DC11C1CC-856B-4A63-823A-2756D8F6B12F

Figs 4, 5, Table 3

###### Type material.

***Holotype***: • male, pinned, with genitalia in a separate microvial. Original label: “Trails in forest, near station at night. -12.2285, -69.1143, 265 M. elev. Finca Las Piedras Res. Station, Madre de Dios. 13-Jul-2024. Coll. E. Emmy F. Medina E., Jared Martin, Johanna Schwartz, Dominic A. Evangelista” “UIRB-PE-23-76”. ***Paratypes***. • 1 adult female: UIRB-PE-25-42. 9 juveniles: AUDE-PE-3-76, UIRB-PE-23-93, UIRB-PE-25-35, UIRB-PE-25-36, UIRB-PE-25-78, UIRB-PE-26-23, UIRB-PE-26-25, UIRB-PE-26-38, UIRB-PE-31-51 (locality and other data for all specimens are given in Table 4).

**Figure 4.**
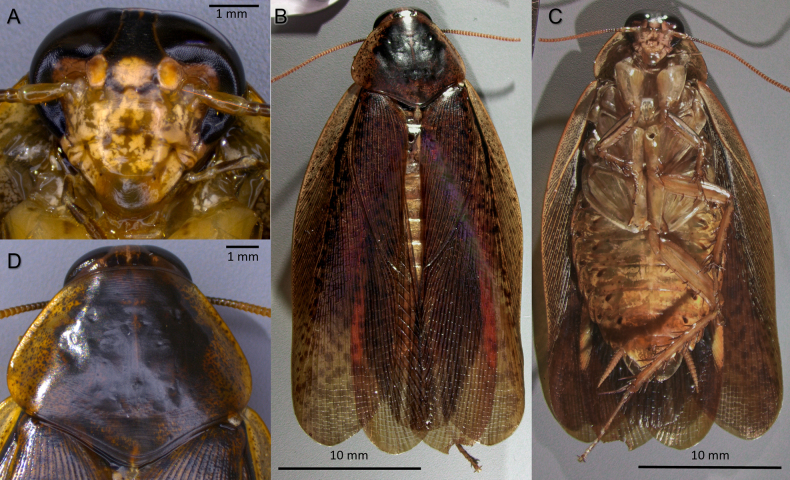
*Epilampra yompori* sp. nov. adult male, holotype UIRB-PE-23-76. **A**. Details of head; **B**. Dorsal habitus; **C**. Ventral habitus; **D**. Details of pronotum. Scale bars: 1 mm (**A, D**); 10 mm (**B, C**).

**Figure 5.**
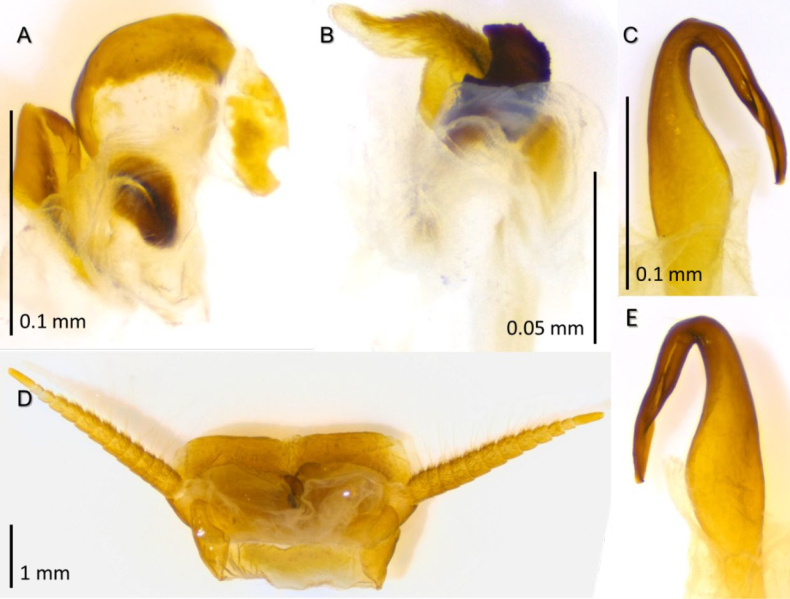
*Epilampra yompori* sp. nov. male genital details, holotype UIRB-PE-23-76. **A**. Dorsal view of R’ phallomere; **B**. *via* and L10’ with apex of *lve*; **C, E**. L3’ with large subapical incision; **D**. ventral view of the dorsal terminal segments. Scale bars: 0.1 mm (**A, C**); 0.05 mm (**B**); 1 mm (**D**).

###### Determination.

This specimen was identified as *Epilampra* (vs *Poeciloderrhis*) based on presence of the following characters: the absence of medial tergal modifications on T1 and T2 (present in *Poeciloderrhis*), hooked phallomere (L3’) with a subapical incision (incision absent in *Poeciloderrhis*), and R’ phallomere cleft not fused (vs fused in *Poeciloderrhis*).

###### Differential diagnosis.

The species may be differentiated from other *Epilampra* spp. by a unique combination of the following characters. The pronotal coloration and *via*+L10’ morphology are most similar to species in the *abdomennigrum* group. However, the species lacks a setal brush on R’ phallomere, placing it in the *burmeisteri* group. Roth (1970d) separated subgroups A and B of the *burmeisteri* group based on the size of *via* compared to L10’ (*via* small in subgroup A, and large in subgroup B). By relative size, this species would fall in subgroup A. However, L10’ is elongated (as in *E. grisea* – *abdomennigrum* group), so *via* may appear small relative to L10’ when in fact it is normally sized when compared to *lve*. Among the species in the *burmeistei* group, the L10’ and *via* shape are most similar to *E. opaca* or *E. columbiana*. The species differs strongly from *E. opaca* in the following characteristics: males 26 mm long (vs 20.5 mm in *E. opaca* (Evangelista et al. 2015)), central region of pronotum fuscous as in *E. taira* (speckled with a symmetrical pattern in *E. opaca* and *E. columbiana*), and L10’ elongated like in *E. grisea* (vs mostly rounded in *E. opaca and E. columbiana*).

###### Remarks.

Roth (1970d) also noted the similarity in *via* and L10’ between various species in the *abdomennigrum* and *burmeisteri* groups. It is possible that these are close relatives, or that one is nested within the other. Hence, we refrain from placing *E. yompori* in either group or creating a new one for it.

###### Description of holotype.

**Male** (UIRB-PE-23-76). ***Head***. Partially visible from dorsal side. Face predominantly pale brown with a dark brown or blackish interocular space ending at the anterior end of the ocelli, except for a circular pale patch in the interocellar space. Ocelli are circular, flat, slightly impressed, and adjacent to the anterior-medial corners of antennal sockets.

***Thorax*. *Pronotum***. Subtrapezoidal anteriorly and with an elongated medial point posteriorly (typical of *Epilampra* spp.). Coloration pale brown near the lateral borders, but mainly dark brown with speckled coloration toward the lateral, and posterior margins. The middle of the pronotum is more solidly dark brown. The thorax in the ventral view is pale brown. ***Legs***. Primarily pale brown but with medium brown (or slightly reddish brown) stripes on the femora, with darker shaded spines on the femora and tibiae. The forefemur AV margin spination is type B2 with four large spines proximal to a row of short spines (16 on the right leg). On the posteroventral margin, there are four large spines, one of which is an apical spine. Foretibia has seven dorsal and five ventral spines and is darker than the femur. All tarsi have five tarsomeres and euplantulae, simple symmetrical claws with arolia. Foretarsus has longer first and fifth tarsomeres, with two rows small proximal spines on ventral side of the first tarsomere. Midfemur with four anteroventral spines, one of which is apical, one anterodorsal apical spine, and four posteroventral spines. The mid-tibia has 15 dorsal spines, two of which are apical, and 12 ventral spines, including one apical spine considerably longer than the other spines on the midtibia. First tarsomere of the middle leg considerably longer than the others with a row of small spines on the ventral side, and two short anterior spines and one short posterior spine. Second tarsomere also has two linear rows of short ventral spines. The first, second, and third tarsomeres have small apical spines on either side of the euplantulae. Hind femur with the same coloration and spination as middle leg. Hind tibia with ten dorsal spines, two of which are apical, and eight ventral spines. Hind tarsus with the first tarsomere slightly larger than all other tarsomeres combined and has two parallel rows of short spines along length of ventral margin and a shorter, more spaced-out row of short spines on the anterior margin. Second tarsomere has two parallel rows of short spines on ventral margin and apical spines around the euplantulae. The third tarsomere also has spines on either side of the euplantulae. Macropterous with tegmina terminating much posterior to the end of the cerci.

***Abdomen***. Base coloration similar to the legs, but with some scant speckled and larger brown spots laterally and a rusty brown region towards the posterior end on the ventral side. Dorsal side without distinctive markings and lacking any tergal specializations. SA plate semi-circular with a short medial division. SG plate with typical *Epilampra* shape (slightly asymmetrical, concave on the right side, with pin-like styli). L3’ strongly curved and narrow, with a very noticeable subapical incision.

###### Range.

We have no data from this species anywhere other than Madre De Dios, where it is widespread.

###### Habitat.

Low hill forest and flooded alluvial forest.

###### Etymology.

The specific epithet *yompori* means “forest spirit” in Piro, an indigenous language in southwestern Amazonia, spoken by communities in Madre de Dios and Ucayali. The language is widespread, as is the cockroach.

### *Epilampra abdomennigrum* group Rehn & Hebard, 1927

#### *Epilampra grisea* (De Geer, 1773)

Fig. 6

**Figure 6.**
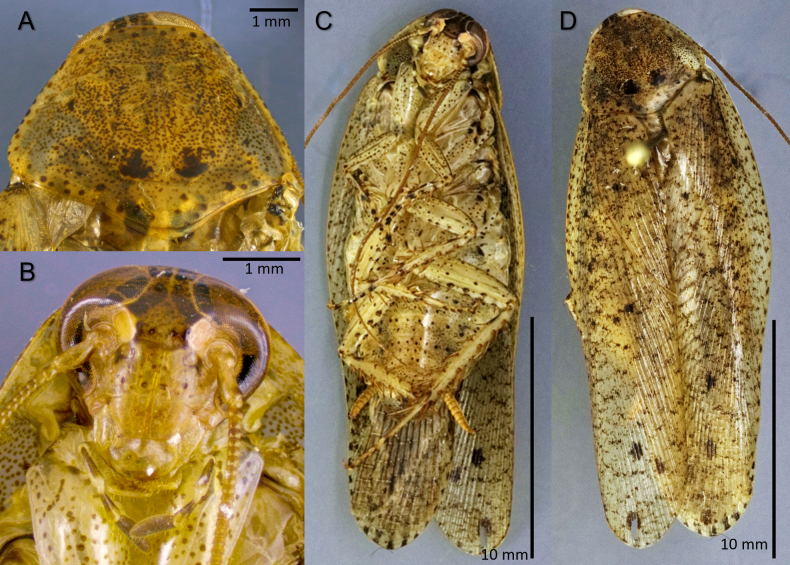
*Epilampra grisea* (De Geer, 1773) adult male, AUDE-PE-1-85. **A**. Details of pronotum; **B**. Ventral habitus; **C**. Dorsal habitus; **D**. Details of head. Scale bars: 1 mm (**A, B**); 10 mm (**C, D**).

**Material examined**. • 2 adult males: AUDE-PE-1-85, UIRB-PE-26-54. 3 adult females: AUDE-PE-3-91, UIRB-PE-24-15, UIRB-PE-26-53 (locality and other data for all specimens are given in Table 4).

**Determination**. This specimen was determined as *E. grisea* based on comparison of male genitalia to those imaged by Roth (1970d).

**Remarks**. This is the first record of this species in Peru.

**Habitat**. Low hill forest and flooded alluvial forest.

### *Epilampra burmeisteri* group Rehn & Hebard, 1927

#### *Epilampra opaca* Walker, 1868

**Material examined**. • Two adult males: UIRB-PE-26-56, UIRB-PE-26-45 (locality and other data for all specimens are given in Table 4).

**Determination**. Color polymorphism in this species was noted previously by Evangelista et al. (2019), and our two specimens match both forms they described. UIRB-PE-26-56 has a paler buffy undertone, and UIRB-PE-26-45 has a darker, more brown-orange undertone (among other minor color differences not worth noting). However, the shape of *via* and the L10’ are very similar, closely matching specimens treated by Roth (1970d).

**Range**. This is the first record of this species in Peru. Within Madre De Dios, this was only collected at Finca Las Piedras Research Station.

**Habitat**. Low hill forest.

##### Epilampra homage

Taxon classificationAnimaliaBlattodeaBlaberidae

Vanker, Medina-Espinoza, & Evangelista sp. nov.

7AF80F3D-A70F-5222-9EDE-CD6AFD603523

https://zoobank.org/25DC1788-66B6-4222-8F3B-BB36A075D676

Figs 7, 8, Table 3

###### Type material.

***Holotype***: • male, pinned, with genitalia in a separate microvial. Original label: “Peru: MD [Madre de Dios], Estación Biológica Los Amigos: Plataforma Trail. 12°33'47"S, 70°06'12"W [-12.565481, -70.1027]. 265 m. 6.vii.2021 [6 June 2021]. night hand coll. D.A. Evangelista leg”. “AUDE-PE-1-88.” ***Paratypes***. • 4 adult males. AUDE-PE-3-64, AUDE-PE-3-63, UIRB-PE-24-14, UIRB-PE-26-70 (locality and other data for all specimens are given in Table 4).

**Figure 7.**
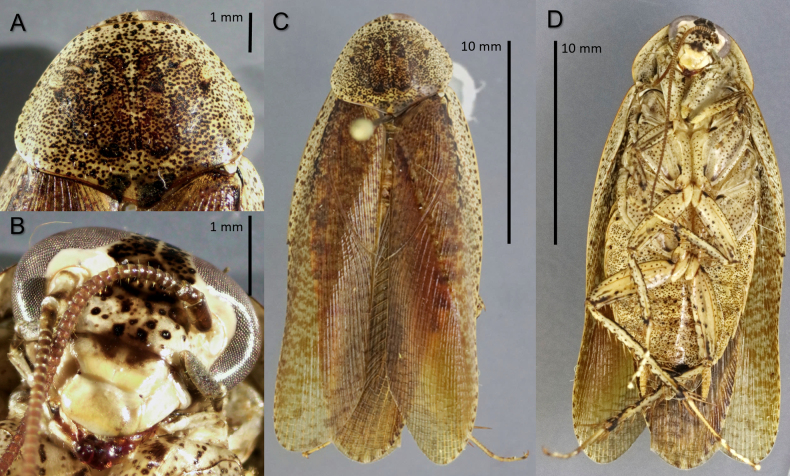
*Epilampra homage* sp. nov. adult male, holotype (AUDE-PE-1-88). **A**. Details of pronotum; **B**. Details of head; **C**. Dorsal habitus; **D**. Ventral habitus. Scale bars: 1 mm (**A, B**); 10 mm (**C, D**).

**Figure 8.**
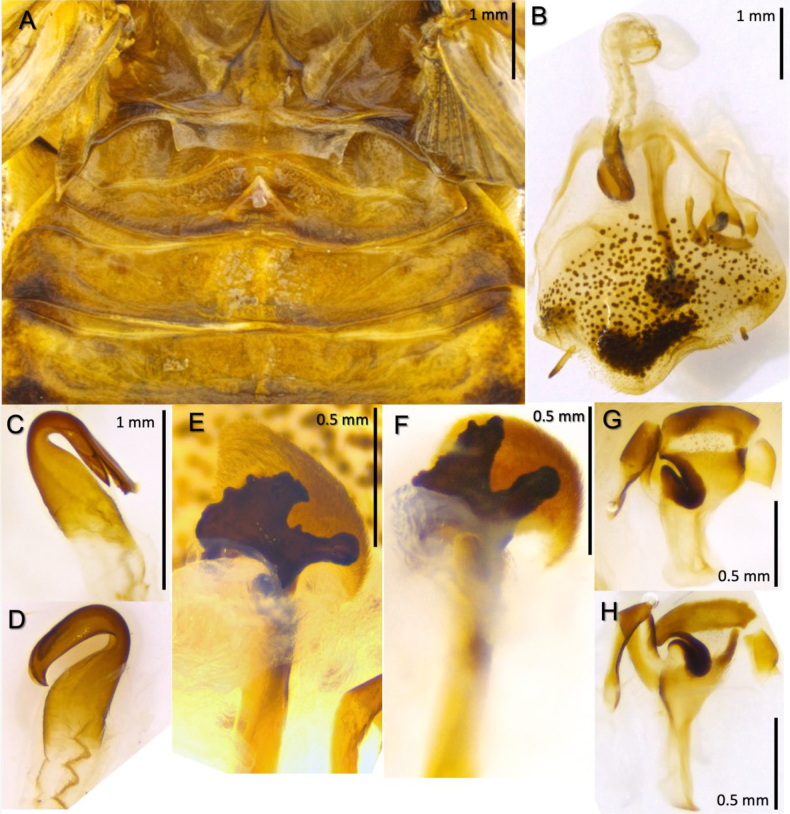
*Epilampra homage* sp. nov. male sexual morphology. **A**. Anterior terga, showing tergal modification on tergum 1 (medial modification and transverse carina with some wide anterio-lateral pits and hairs); **B**. Ventral view of sub-genital plate with genitalia in place; **C**. L3’ with large subapical incision; **D, E**. *via* and L10’ with apex of *lve*; **F**. Dorsal view of R’ phallomere; **G**. Ventral view of R’phallomere. Specimens imaged: (**A, C, D, F, G**) holotype AUDE-PE-1-88 and (**B, E, H**) paratype AUDE-PE-3-64. Scale bars: 1 mm (**A–C**); 0.5 mm (**E–H**).

###### Determination.

This specimen is placed in *Epilampra* (vs *Poeciloderrhis*) based on the presence of the following characters that differentiate *Epilampra* from *Poeciloderrhis*: *via* not pointed (pointed in *Poeciloderrhis*), *via* and *lve* separated (vs fused in *Poeciloderrhis*), hooked phallomere (L3’) with a subapical incision (incision absent in *Poeciloderrhis*), and R’ phallomere cleft not fused (vs fused in *Poeciloderrhis*). However, this specimen does have a modified T1 with a triangular medial knob, which was previously only found in *Poeciloderrhis*. Placement in the *burmeisteri* species group is supported by autapomorphies in the male genitalia (see below).

###### Differential diagnosis.

This species’ male genitalia are almost identical to *E. substrigata*. There are some minor differences in *via* compared to the *E. substrigata* specimens shown by Roth (1970d). Without more spatial sampling it is unclear whether the differences in *via* are distinctive. These species are separated by the presence of a tergal modification on T1 in *E. homage*. *E. homage* also has a transverse carina on T1, but this may be a plesiomorphy as it is found in *Africalolampra*, *Parapoeciloderrhis*, and perhaps other *Epilampra* species. *Epilampra homage* is easily separated from *Africalolampra*, and *Parapoeciloderrhis* in the morphology of L2’: *via* separated from *lve* (vs connected), and the shape of *via* is with an irregular ridge apically (like a mountain range).

###### Description of holotype.

**Male** (AUDE-PE-1-88). ***Head***. With large overlapping speckles, densest between the compound eyes, but the ecdysial suture lacks speckles. Dark band connecting ocelli in a U-shape, and underneath it, the speckles are sparser. Clypeus with some brown coloration at the base (dorsal) but otherwise lacking speckles along with the labrum.

***Thorax*. *Pronotum***. Speckled mostly homogenously throughout but densest medially. Other spots form a panther-face pattern. AV margin of forefemur type B2 with five large spines preceding the row of small spines. On all legs, tarsal claws large, simple, with large arolia and all euplantulae present.

***Abdomen***. Ventral abdomen lightly colored with scattered spots of medium density. SG plate with a with a brown patch on the distal third (but brown patch does not meet the posterior margin). SG plate shape and styli typical of *Epilampra*. T1 with a transverse carina and a medial modification. Medial modification strongly triangular (almost equilateral), with some hairs originating near the carina pointing towards the anterio-lateral sides of the triangle. Hairs line the depression that houses the triangle, which becomes very narrow at its deepest point. Face of triangle concavely dimpled. T1 also modified with shallow depressions on either side, anterior to the carina and aligned with the edges of the square projection of the metanotum. Genitalia as shown in Fig. 8.

###### Remarks.

None of the several female *Epilampra* morphotypes in our collection are obviously attributable to this species. Perhaps some are *E. homage* females but sexual dimorphism (e.g., female *Epilampra* are typically larger than males) is making it difficult to associate the sexes.

###### Range.

We have no data for this species outside of Madre De Dios. Within Madre De Dios, this species was only found at Los Amigos Biological Station, and Finca Las Piedras Research station.

###### Habitat.

Low hill forest.

###### Etymology.

The specific epithet *homage* is meant to imply that this species has a combination of characters present in other taxa (*E. substrigata*, *Poeciloderrhis*, *Africalolampra*, and *Parapoeciloderrhis*), as if it were “paying homage” to them.

##### Epilampra wandpero

Taxon classificationAnimaliaBlattodeaBlaberidae

Medina-Espinoza, Vanker, & Evangelista sp. nov.

990A0DAD-B48F-57D5-B1C5-50053A4FE72E

https://zoobank.org/42B7178A-B313-420D-80ED-F1A1AB0F7EF3

Fig. 9, Table 3

###### Type material.

***Holotype***: • male, pinned, with genitalia in a separate microvial. Original label: “Peru: Madre de Dios, Tambopata, Ecoaventuras Amazonicas, 12°30'20"S, 69°10'26"W [-12.505556, -69.173889], 29.vi.2021 [29 June 2021], night on forest floor. hand coll. D.A. Evangelista leg”. AUDE-PE-03-86. ***Paratypes***. • 16 adult males: AUDE-PE-2-85, AUDE-PE-3-70, AUDE-PE-3-72, AUDE-PE-3-80, AUDE-PE-3-90, UIRB-PE-23-86, UIRB-PE-23-94, UIRB-PE-23-95, UIRB-PE-23-96, UIRB-PE-23-98, UIRB-PE-24-01, UIRB-PE-26-42, UIRB-PE-26-50, UIRB-PE-26-51, UIRB-PE-26-55, UIRB-PE-26-71. 5 adult females: UIRB-PE-26-41, UIRB-PE-26-43, UIRB-PE-26-49, UIRB-PE-26-52, UIRB-PE-26-72 (locality and other data for all specimens are given in Table 4).

**Figure 9.**
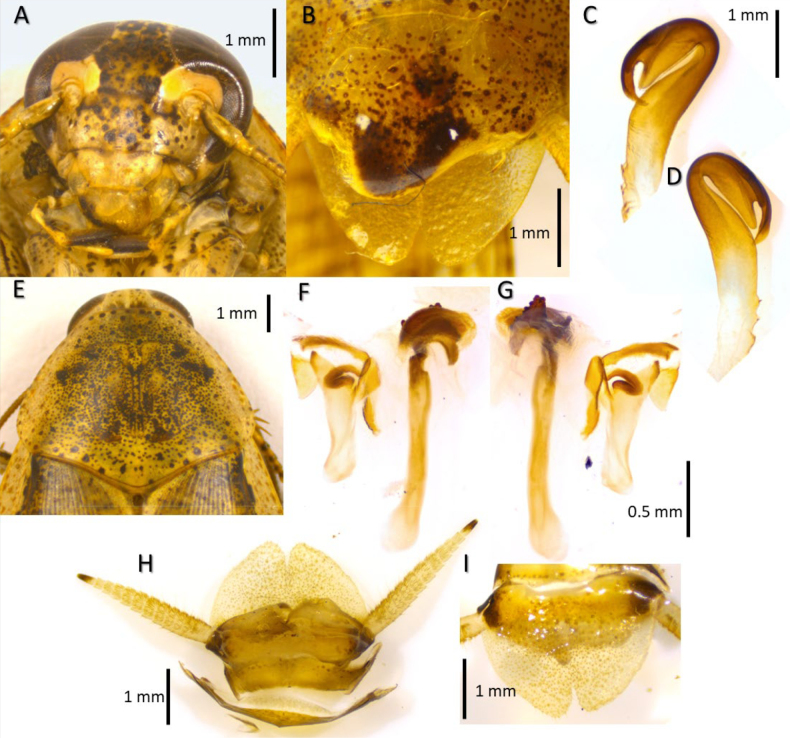
*Epilampra wandpero* sp. nov. adult male morphology. **A**. Details of head; **B**. External details of subgenital plate; **C**. Dorsal view of L3’; **D**. Ventral view of L3’; **E**. Details of Pronotum; **F**. Ventral view of R’ phallomere and L2’; **G**. Dorsal view of R’ phallomere and L2’; **H**. Ventral view of supra-anal plate and paraprocts; **I**. Dorsal view of supra-anal plate. Specimens imaged: (**A, B, E, F, G, H, I**) holotype AUDE-PE-3-86 and (**C, D**) paratype UIRB-PE-24-01. Scale bars: 1 mm (**A, B, C, D, E, H, I**); 0.5 mm (**G**).

###### Determination.

We identified this taxon as *Epilampra* based on the mottled coloration, medio-posterior lobe on pronotum, elongated cerci (compared to stout in most other Blaberidae), separated *via* and *lve*, and lack of a visible tergal gland in males. We could not associate any juveniles with adults based on morphology. We tentatively associated males and females based on some similarities in color pattern. *Epilampra wandpero* was determined to be in Roth (1970d)’s subgroup B based on the large *via* covering most of L10’.

###### Differential diagnosis.

The new taxon can be differentiated from all other *Epilampra* spp. treated by Roth (1970d) by the shape of L3’, in which the hook is enlarged, with a very large sub-apical incision, giving the hook a crab-claw-like appearance. In other *Epilampra* spp. with a robust hook, the smaller lip of the hook (i.e., the inner side of the sub-apical incision) is still slender. It is most closely related to *E. campestris* (Rocha e Silva and Auguiar 1978) based on the similarity of the SA plate, *via*, and L10’. Yet, the two species differ strongly in the shape of L3’, pronotal coloration, and head coloration.

###### Description of holotype.

**Male** (AUDE-PE-03-86). ***Head***. Two ocelli flat and distinctly formed; vertex pale brown with small dark spots; frons pale brown with small dark spots in greater density between the antenna than under the antenna, darker brown band above the epicranial suture fading as it approaches the vertex, with a horizontal thin dark brown band connecting the inferior side of the ocelli; dark brown line in the epistomal and subgenal sulcus not meeting in the middle; labrum yellowish with pale brown areas in the lateral sides and its inferior side; maxillary palps yellowish: first segment with an apical pale-brown ring; second with a dark brown area in ventral side, third yellowish with dark brown ventral side, and fourth dark brown.

***Thorax*. *Pronotum***. Trapezoidal with a rounded anterior edge and a bell-curve shaped posterior edge (typical of Epilamprinae). Pale brown, scattered maculations, with a defined row of dark brown spots on the anterior edge. After the anterior-most quarter of the pronotum, two lateral lines strongly curved posteriorly towards the midline without intersecting and ending shortly after the curve. Two thin parallel dark brown lines in the center of the pronotum, anteriorly curved gently outward; beginning in the posterior quarter, the lines separate, and two parallel lines appear between them, resembling a fork. Mesothorax and metathorax are not visible from dorsal view. Thorax in ventral view pale brown with dark brown spots.

***Legs***. Forelegs: forecoxae with small dark brown spots, fore-femoral pale brown, dark brown lines in the edges, five large anteroventral spines towards the proximal side, followed by a row of 14 short spines and two longer spines; four posteroventral spines; one genicular spine. Foretibia pale brown, not uniformly slender, expanding distally, with setae in the anteroventral side, ten strong spines distributed around the tibia, anteroventral side dark brown, distal end with a dark brown ring. Tarsi with five tarsomeres, the first one elongated with a bulbous end, two rows of tiny spines in the anteroventral side, and one apical spine; the next three moniliform with a spine in the distal end, the second one with an apical spine. Mid-legs missing due to damage. Hind-legs: hind coxae with small dark brown spots; hind femoral pale brown with small dark brown spots, with lines along its dorsal and ventral edges; four spines in the anteroventral side and three in the posteroventral side. Foretibia, pale brown with dark brown ventral edge, thirteen large spines on the dorsal side, nine large spines at the ventral side, one large genicular spine, and one short apical spine, distal end with a dark brown ring. Tarsi with five tarsomeres; first and second tarsomeres cylindrical with two parallel rows of tiny spines on the ventral side, the first one the largest; third tarsomere shorter with two tiny spines on the ventral view; a couple of apical spines on each side of the distal border; and fourth tarsomere short without spines; the fifth tarsomere longer without spines. Claws simple, symmetrical, arolium present. Euplantulae present on all tarsomeres. ***Wings***. Longer than the abdomen. Forewing dark-brown, pale brown in the costal area of the wing, a row of black dots roughly following the radial vein until midway towards the tip, marks a sudden transition to the darker tan.

***Abdomen***. Pale brown, covered with dark brown dots, increasing in density to the posterior side, a row of larger dark brown dots in the distal margin. No visible external tergal glands, supra-anal plate bilobed. Subgenital plate shape typical for *Epilampra*, with slender styli.

***Genitalia***. L3’ long and slender, with tip strongly curved acutely with a very large sub-apical incision, with the inner portion truncated and bulbous, giving the appearance of a crab-like claw. *via* large and covering most of L10’, similar to *E. opaca*, *E. substrigata*, and *E. columbiana* in Roth (1970d). R’ phallomere like *E. columbiana* but narrower with cleft deeper.

###### Remarks.

Two paratype males with facial coloration pattern like the holotype. Three paratype males with the spots in the center of the frons extend from the inner edge of the eyes, almost meeting in the center. Paratypes show variation in color intensity and spot density on the pronotum.

###### Range.

We have no data for this species outside of Madre de Dios. Within the region, it was most common at Kawsay Biological Station, and present at all other sites except Finca Las Piedras Research Station.

###### Habitat.

Low hill forest and flooded alluvial forest.

###### Etymology.

The specific epithet means cockroach in Harakmbut, the language of the indigenous Harakbut ethnic people living in the Madre De Dios region.

#### Epilampra sp. cf. azteca “MDD”

Fig. 10, Table 3

**Material examined**. • 1 adult male: AUDE-PE-3-96. 1 adult female: AUDE-PE-3-92 (locality and other data for all specimens are given in Table 4).

**Figure 10.**
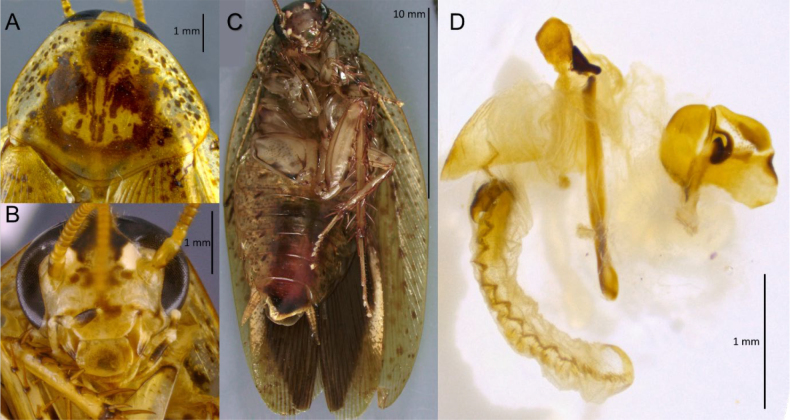
*Epilampra cf. azteca* “MDD” male, AUDE-PE-3-96. **A**. Details of pronotum; **B**. Details of head; **C**. Ventral habitus. **D**. Male genital phallomeres. Scale bars: 1 mm (**A, B, D**); 10 mm (**C**).

**Determination**. The genitalia of this specimen (particularly the shape of *via* and L10’) strongly correspond to the mainland South American forms of *E. azteca* as reported by Roth (1970d). However, given the variation in *via* and L10’ within *E. azteca* (Roth 1970d), and between *E. azteca* and *E. rothi* (Fisk and Schal 1981) we are not confident in calling this specimen *E. azteca*. Fisk and Schal (1981) and Roth (1970d) all noted other subtle variation in coloration and L10’ morphology. The taxonomic value of these variations is unclear without comparing specimens from a broader region. As such, we refrain from assigning a specific identification or describing this as a new species.

**Range**. Within Madre De Dios, this taxon was only collected at Los Amigos Biological Station.

### Epilampra mexicana group Rehn & Hebard, 1927

#### *Epilampra conferta* Walker, 1868

Fig. 11

**History**. Roth (1970d) discusses disagreement by other taxonomists and his own lack of clarity on the status of *E. conferta*. Roth (1970d) discussed variation among specimens identified as *E. conferta* leading him to state “*Epilampra conferta* may well be a complex of sibling species”. This situation likely cannot be resolved without genetic information since the type specimen is a female (Walker 1868).

**Figure 11.**
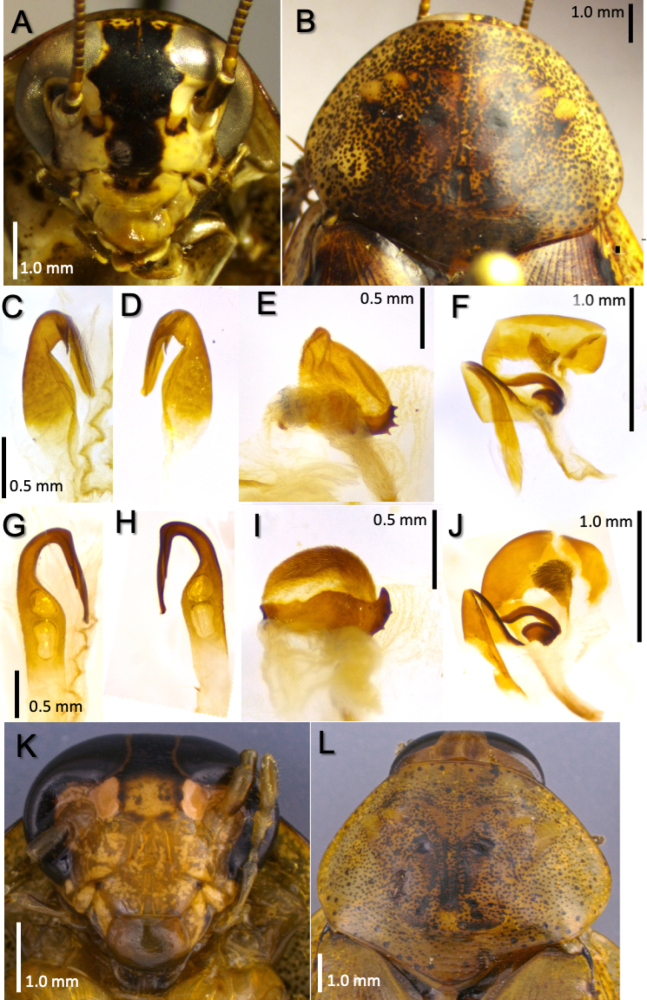
*Epilampra conferta*. **A–F**. *Epilampra conferta* “barra” male; **G–L**. *Epilampra conferta* “placa” male; **A, K**. Head coloration differs in the pattern and extent of black pigmentation; **B, L**. Pronotal pattern is almost identical between the two types, and variation within each type (not shown) is also present; **C–J**. Morphology of the male genital sclerites (**C, D, G, H**) L3’, (**E, I**) L10’+L2’ and (**F, J**) R’ in dorsal view show clear differences, but it is unclear which (if either) of these forms more closely corresponds to the female type of *E. conferta*. Specimens imaged: (**A–F**) AUDE-PE-1-67 and (**G–L**) UIRB-PE-26-48. Scale bars: 1 mm (**A, B, F, J, K, L**); 0.5 mm (**C, E, J, I**).

**Remarks**. We think *Epilampra conferta* is comprised of more than one species. Each of the species discussed below is most similar to *E. conferta* as figured by Roth (1970d). Roth (1970d: figs 24–35) showed a series of morphologically variable specimens from Central America and Ecuador that are most similar to specimens we refer to as *Epilampra conferta* “placa” below. Roth (1970d: figs 36–46) showed morphologically variable specimens identified as *E. conferta* from Peru, Ecuador, and Colombia, which are quite different from *Epilampra conferta* “placa”. Roth (1970d: figs 36–43) are most similar to the specimens we refer to as *Epilampra conferta* “barra”. Thus, representatives of two morphotypes presented by Roth (1970d) are sympatric in Madre de Dios and are morphologically distinct. Unfortunately, images of the type on Cockroach Species File (BMNH (E)#878063) show a cockroach with significantly different coloration (head, pronotum, and abdomen) than any of our specimens.

To summarize, *Epilampra conferta* “placa” and *Epilampra conferta* “barra” are distinct species based on multiple morphological characters described below. Each is likely to be distinct from *Epilampra conferta* Walker, 1868, but we cannot demonstrate this due to lack of genetic data. We do not erect a new species name for any of these since we cannot know which set, if any, corresponds to the type of *E. conferta*. Erecting new names for one, or both, of the taxa below could create additional taxonomic confusion. We also do not want to describe these as subspecies since they are clearly distinct from one another.

#### *Epilampra conferta* “placa”

Fig. 11A–F

**Material**. Males: UIRB-PE-24-06, UIRB-PE-26-80, UIRB-PE-26-44, UIRB-PE-24-02, UIRB-PE-24-04, UIRB-PE-24-05, UIRB-PE-23-97, UIRB-PE-24-03, UIRB-PE-26-48. AUDE-PE-1-74, AUDE-PE-1-86, AUDE-PE-3-68, AUDE-PE-3-69, AUDE-PE-1-84, AUDE-PE-3-75, AUDE-PE-3-87, AUDE-PE-3-74, AUDE-PE-3-59, AUDE-PE-3-68. Females: UIRB-PE-24-49, UIRB-PE-24-07, UIRB-PE-24-11, UIRB-PE-23-77, UIRB-PE-23-80, UIRB-PE-23-99, UIRB-PE-, AUDE-PE-03-79, AUDE-PE-01-87 (locality and other data for all specimens are given in Table 3).

**Determination**. This taxon was placed in *Epilampra* (vs *Poeciloderrhis*) based on the presence of the following characters: absence of medial tergal modifications on T1 and T2 (present in *Poeciloderrhis*), *via* separated from *lve* by a membrane (connected in *Poeciloderrhis*), hooked phallomere (L3’) with a subapical incision (incision absent in *Poeciloderrhis*), and R’ phallomere cleft not fused (vs fused in *Poeciloderrhis*). This species was identified as *mexicana* group based on the genital features given in the key, in particular, the shape *via* (L2’) and its connection to L10’.

**Remarks**. Comparison of male genitalia to Roth (1970d: figs 24–35) facilitated determination. Prior to dissection, we had separated this species into at least four morphotypes based on color and size polymorphism. All specimens share a similar pronotal pattern but there is huge variability in the intensity of colors. For instance, AUDE-PE-1-82 has very dense speckling around the robust panther-face pattern, whereas AUDE-PE-1-86 has delicate speckling, and some pale buff “eyebrow-like” marks on the panther-face. Morphological measurements are presented in Table 4.

**Range**. This was among the most common Blaberidae in Madre De Dios, Peru and was widespread throughout the region.

**Habitat**. Low hill forest and flooded alluvial forest.

#### *Epilampra conferta “*barra*”*

Fig. 11G–L,19

**Material**. Males: AUDE-PE-1-67, AUDE-PE-1-82, AUDE-PE-2-66, AUDE-PE-2-94, AUDE-PE-2-64, AUDE-PE-2-63, AUDE-PE-2-61, UIRB-PE-26-46 (locality and other data for all specimens are given in Table 3).

**Determination**. This taxon was placed in *Epilampra* (vs *Poeciloderrhis*) based on presence of the following characters: the absence of medial tergal modifications on T1 and T2 (present in *Poeciloderrhis*), *via* separated from *lve* by a membrane (connected in *Poeciloderrhis*), hooked phallomere (L3’) with a subapical incision (incision absent in *Poeciloderrhis*), and R’ phallomere cleft not fused (vs fused in *Poeciloderrhis*). This specimen was identified as the *mexicana* group of *Epilampra* based on the genital features given in the key, particularly *via* (L2’) shape and its connection to L10’.

**Differential diagnosis**. This taxon is most similar specimens figured by Roth (1970d) that had been previously identified as *E. conferta* but demonstrated significant variability (e.g., Roth, 1970b: figs 36, 39, which are also from Peru). This taxon is similar to *E. conferta “*placa*”* but differs strongly in the shape of *via* (L2’). In *E. conferta “*placa*”*, *via* is plate-like and protrudes from L10’ on the side bearing teeth (e.g. Roth 1970d: figs 27, 31, 32, 34) and lacks a strong plate-like extension on the opposite side. In *E. conferta* “barra”, *via* is more bar-like, the side bearing teeth does not extend much past the edge of L10’ (if at all), and bears a plate-like extension on the opposite side. In *E. conferta* “barra” L10’ is not distinctly shaped (*E. conferta* “placa”’s L10’ is shaped like the back of a chair). Also, in comparing only among our specimens, *E. conferta* “barra” has more black color of the frons (*E. conferta* “placa” has some black between the eyes but pale-colored on the lower part of the frons).

**Remarks**. We have only identified males of this species, in contrast to *Epilampra conferta* “placa” where we have both males and females. Among numerous unidentified female-only morphotypes, none are obviously *Epilampra conferta* “barra”. One lone female (UIRB-PE-23-79) has the dark-colored frons, but is brachypterous and much more melanized overall. All other female morphotypes either have a different pronotal pattern or are lacking the black frons. Morphological measurements are presented in Table 4.

**Habitat**. Low hill forest.

#### *Epilampra sodalis* group Roth (1970d)

##### Epilampra sofia

Taxon classificationAnimaliaBlattodeaBlaberidae

Evangelista, Medina-Espinoza, & Vanker sp. nov.

397E99A1-B192-5D86-867C-E8D1895BB855

https://zoobank.org/C0E4E1A5-F818-4CA5-B51F-DDB32D6355A0

Figs 12, 13

###### Type material.

***Holotype***. • Male, pinned, with genitalia in a separate microvial. Original label: “within concession, near station at night. -12.5282, -69.0141. 175 M elev. Kawsay Biol. Station, Madre de Dios, Peru. 18-Jul-2024|Coll: Emmy F. Medina E., Jared Martin, Johanna Schwartz, Dominic A. Evangelista [leg].” UIRB-PE-26-37. ***Paratype***. • Female: UIRB-PE-25-77 (locality and other data for all specimens are given in Table 3).

**Figure 12.**
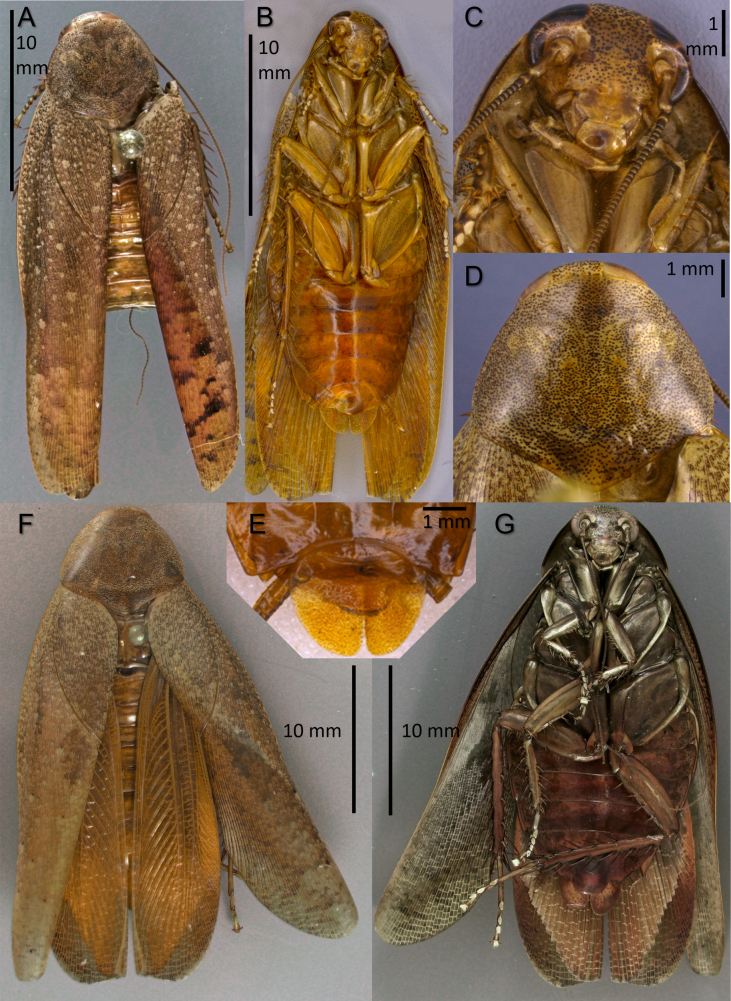
*Epilampra sofia* sp. nov. external morphology. **A**. Dorsal habitus of male; **B**. Ventral habitus of male; **C**. Head; **D**. Pronotum; **E**. Dorsal terminalia highlighted; **F**. Dorsal habitus of female; **G**. Ventral habitus of female. Note: The subtle orange coloration of the posterior end of this species may not be perfectly captured in all reproductions (or all screens, of view digitally) of this manuscript. Also, while the anterior of the specimen in (**G**) appears black, in reality it is more of a very dull brown-grey. Specimens imaged: (**A–E**) holotype UIRB-PE-26-37, and (**F, G**) paratype UIRB-PE-25-77. Scale bars: 10 mm (**A, B, F, G**); 1 mm (**C, D, E**).

**Figure 13.**
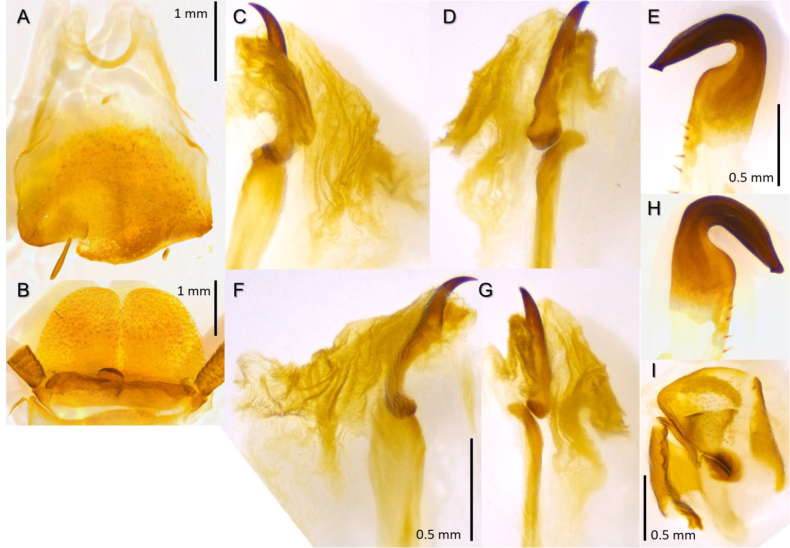
*Epilampra sofia* sp. nov. male sexual morphology, holotype UIRB-PE-26-37. **A**. Ventral view of dissected subgenital plate; **B**. Ventral view of dissected supra-anal plate and paraprocts; **C, D, F, G**. *via*, L10’, and apex of *lve* from various angles; **E, H**. L3’ hooked phallomere; **I**. Dorsal view of R’ phallomere. Scale bars: 1 mm (**A, B**); 0.5 mm (**F, E, I**).

###### Determination.

The specimen is nearly identical to *E. sodalis* in the literature (Roth 1970d; Anisyutkin 2016) except for the L10’, *via*, and L2’ cleft.

###### Differential diagnosis.

This is a distinct species from *E. sodalis* and *E. carsevennae* (Bonfils 1975) based on the following three characters. L10’ large, enveloping most of *via* and covered with weakly pigmented microtrichia (vs *E. sodalis*’ and *E. carsevennae*’s L10’ which are indistinct or entirely reduced). *via* with a unique shape (lacking the medial knob figured in *E. sodalis* and not hook-shaped like in *E. carsevennae*). Finally. The R1’ cleft is fully fused, which Roth showed was the case in some *sodalis* group representatives, but usually only partially. R1’ in *E. carsevennae* is not well-illustrated.

###### Description of holotype.

**Male** (UIRB-PE-26-37). ***Head***. Shape and proportions of head as in Anisyutkin (2016: fig. 6) Frons speckled, almost homogenously, but speckles lacking on the clypeus and labrum. Chestnut colored spots (oval shaped) slightly under ocelli and slightly smaller than the ocelli.

***Thorax***. Speckled almost homogenously throughout pronotum, with small areas with larger areas of negative space forming a symmetrical pattern similar to the panther-face pattern on other species (e.g., *E. conferta*). Speckles larger than *E. sodalis* in Evangelista et al. (2015) and *E. carsevennae*. AV margin of foreleg with type B2 spination (three large spines at base). All four euplantulae very large on all tarsomeres (four on each leg). Tarsomere spination typical of *Epilampra*, with small spines on either side of euplantulae and two rows of small spines at base. Coxa colored like pronotum and head, but with smaller speckles. Other leg segments similarly colored but lacking speckles. Base color of tegmina a pale, whitish brown, with darker brown highlights on the cell and vein borders. Whitish brown patches lacking other pigmentation throughout the exposed portion of the tegmina, getting larger distally. Portion of right tegmina that overlaps with left tegmina colored distinctly differently, with an orange-brownish base color and large dark-brown spots.

***Abdomen***. Abdomen reddish-orange with heterogeneous (in size and distribution) speckles. SG plate typical of Epilamprinae (two simple, subsymmetrical styli with the right one emerging from a concave asymmetry in the margin). SA plate symmetrical and large, the preceding tergite with a large convexity medially, spanning almost the entire width of the SA plate. Genitalia as shown in Fig. 13. Morphological measurements are presented in Table 4.

###### Description of female paratype.

**Female** (UIRB-PE-25-77). Same as male except larger and with the following departures. Hind legs with only three tarsomeres. Ventral coloration is slightly different, with the thorax having more prominent brown-grey coloration, and the abdomen having a much stronger reddish coloration with an almost purple-pink tinge.

###### Remarks.

We have no data for this species outside of Madre De Dios, Peru. Within the region, we collected only two adult individuals, both from Kawsay Biological Station.

###### Habitat.

Flooded alluvial forest.

###### Etymology.

The specific epithet, *sofia*, in apposition, is named after the daughter of DAE.

### *Epilampra paititi* group

**Differential diagnosis**. This is similar to the *sodalis* species group in that both *Epilampra sodalis* “sp. A” (Roth 1970d) and this taxon have modified T3 and T4, but they differ hugely in the shape of *via* and L10’: *via* is flatter and wider (not claw-shaped) and is strongly fused to L10’, which has a plated sclerite and extensive microtrichia of two types: fine and thick. It is also similar to the *mexicana* group in the having *via* strongly fused to L10’ and bearing a plated sclerite, but they differ in the tergal gland modification (absent in *mexicana*), absence of the setal brush (present in *mexicana*), and details of *via* (species in the *mexicana* group have only one type of microtrichia). The thick microtrichia are localized to the part of L10’ that is on the opposite side from *via* and forming a bar-like ridge. The *paititi* group is also similar to the *Huesseriana* group in *via* being fused to L10’ and the rounded shape of L3’ but differs in L10’ bearing extensive microtrichia of two types (vs one) and having a plated sclerite. This taxon also differs from all the above species groups by the posterior edges of terga 1–5 being slightly upturned (vs not being upturned).

**Description**. *via* is clearly separated from *lve*, L10’ has two types of microtrichia as described above, and part of L10’ is not formed into microtrichia but in the (plesiomorphic) sclerite form. L3’ is very long when extended, and the setae on the basal lumps are present. The apical portion of L3’ forms a rounded, curved hook with a very small sub-apical incision. R’ phallomere is lacking the setal brush, and the cleft is not fused together. T3 is modified with a longitudinal medial hump (vs no medial hump, or modifications on T3 and T4), and T4 has a wide, shallow basin medially.

#### Epilampra paititi

Taxon classificationAnimaliaBlattodeaBlaberidae

Vanker, Medina-Espinoza, & Evangelista sp. nov.

8A63D48B-9F38-5490-B356-AE4435A56311

https://zoobank.org/EC9318CA-D95B-414D-B91B-65A7DB6B76F5

Figs 14, 15

##### Type material.

***Holotype***. • Male, pinned, with genitalia in a separate microvial. Original label: “Peru: Madre de Dios, Estación Biológica Los Amigos. Plataforma trail, terra firme; 12°33'47"S, 70°06'12"W [-12.563056, -70.103333]; 3.vii. 2021 [3 July 2021], hand coll. D.A. Evangelista leg.” AUDE-PE-1-83.

**Figure 14.**
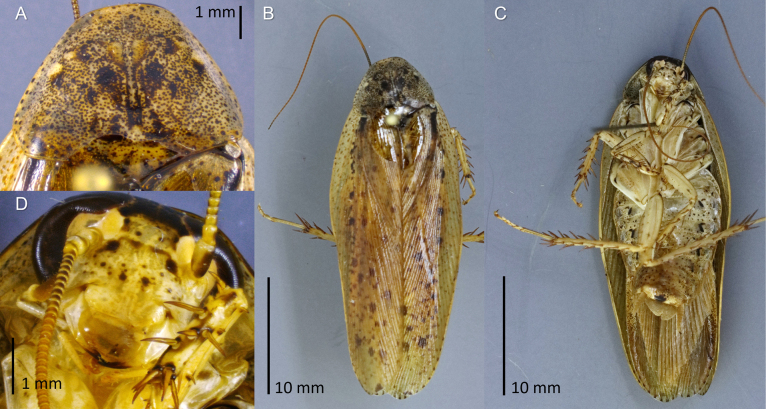
*Epilampra paititi* sp. nov. male, holotype (AUDE-PE-1-83). **A**. Pronotum; **B**. Dorsal habitus; **C**. Ventral habitus; **D**. Head. Scale bars: 1 mm (**A, D**); 10 mm (**B, C**).

**Figure 15.**
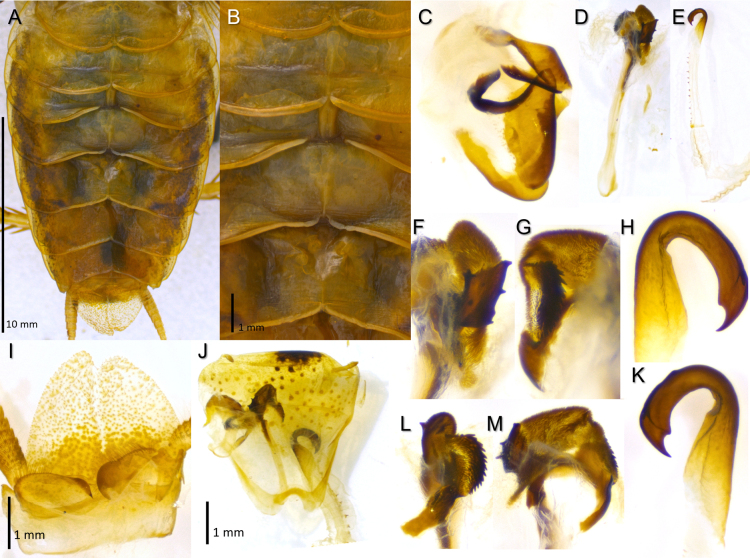
*Epilampra paititi* sp. nov. male sexual morphology, holotype (AUDE-PE-1-83). **A, B**. Dorsal tergal modification; **C**. Dorsal view of R’ phallomere; **D**. L2’; **E**. Dorsal view of L3’. **F, G, L, M**. *via* and L10’ from various angles; **H**. Ventral view of hook (hla) of L3’; **K**. Dorsal view of hook (hla) of L3’; **I**. Ventral view of supra-anal plate and paraprocts; **J**. Ventral view of sub-genital plate and genitalia in place. Scale bars: 10 mm (**A**); 1 mm (**B, I, J**).

##### Determination.

This specimen was placed in *Epilampra* (vs *Poeciloderrhis*) based on the presence of the following characters: the absence of medial tergal modifications on T1 and T2 (present in *Poeciloderrhis*), *via* separated from *lve* by a membrane (connected in *Poeciloderrhis*), hooked phallomere (L3’) with a subapical incision (incision absent in *Poeciloderrhis*), and R’ phallomere cleft not fused (vs fused in *Poeciloderrhis*). We could not associate any females or juveniles to the adult male based on morphology.

##### Differential diagnosis.

At first, this species seemed to belong in the *sodalis* group based on the following traits it shares with taxa in that group: L10’ is solidly fused to *via* and covered in microtrichia (shared with Roth (1970d)’s *Epilampra* sp. C), and presence of a medial tergal modification on T3 and T4 (shared with Roth (1970d)’s *Epilampra* sp. A). In fact, Roth (1970d) commented that the latter of these traits was the only known cockroach with such a tergal gland. We do not know if this remains true outside of *Epilampra*. Yet, *Epilampra* sp. A and now this new species are unique within *Epilampra* in having such a dorsal tergal modification. However, this species differs strongly from *Epilampra* sp. A in the shape of all the genital sclerites (including L10’), although L3’ is more-or-less similar in the two taxa. The new species also differs strongly from *Epilampra* sp. C in the shape of *via* and L1.

##### Description of holotype.

**Male** (AUDE-PE-1-83). ***Head***. Frons, in dorsal view, predominantly pale brown with some dark brown speckling above the anteclypeus and a dark patch in the interocular space. Ocelli are flat, slightly ovular, and adjacent to the anterior-medial corners of antennal sockets. Labrum and final segment of maxillary palp slightly darker than the pale brown color of most of the face.

***Thorax***. Subtrapezoidal anteriorly, with an elongated medial lobe posteriorly. Primarily pale brown with dark speckles and several dark brown patches towards the midline. In the center are two dark parallel lines. Forewings large, extending past the end of abdomen and cerci. Pale brown coloration, speckled across wings with fainter and smaller speckles towards the lateral and posterior margins of the wings. Coloration on all legs similar, primarily pale brown with some sparse speckling that is most dense on the coxa, and a dark brown line on the dorsal margin of all femurs. Forefemur with a dark brown line on anterior margin. The forefemur’s AV margin spination type is B2 with five large spines proximal to a row of 19 short spines. Forefemur posteroventral margin with four spines, one of which is apical. Fore and hind tarsus with five tarsomeres, and midtarsus with four tarsomeres. Fore- and midtarsus with two parallel rows of short ventral spines on the first tarsomere, short spines on either side of the euplantulae, and a final tarsomere with simple symmetrical claws and an arolium. Hind tarsus damaged without the final clawed tarsomere absent.

***Abdomen***. Dorsal abdomen has pale tan coloration at the anterior end of the abdomen, which darkens towards the posterior end, and light speckling on the left and right margins. Tergal modification on T3. Posterior margin of T1–5 medially notched. Ventral abdomen is pale brown and speckled. SA plate is semi-elliptical and bilobed with a long medial division. SG plate typical of *Epilampra* with short straight styli and marginal concavity on the right. Genitalia as in Fig. 15. Morphological measurements are presented in Table 4.

##### Range.

We collected only a single adult male of this species (if we collected an immature, or female, we have not identified it) in Madre De Dios, Peru, and we have no data for this taxon outside the region. We found it only at Los Amigos Biological Station.

##### Habitat.

Low hill forest.

##### Etymology.

The specific epithet *paititi* draws a parallel between the mythological city of gold lost in Madre De Dios and the now discovered “lost tergal gland of gold”.

### Zetoborinae Burmeister, 1838

***Phortioeca* Saussure, 1862**

#### *Phortioeca* sp.

**Material**. UIRB-PE-20-37, UIRB-PE-20-38, UIRB-PE-20-39, UIRB-PE-20-40, UIRB-PE-20-41, UIRB-PE-20-42, UIRB-PE-20-43, UIRB-PE-20-44, UIRB-PE-20-45, UIRB-PE-26-59 (locality and other data for all specimens are given in Table 3).

**Determination**. We identified this species as *Phortioeca* in the field based on the large size, distinctive coloration of wings and pronotum, and the distinctive pronotal shape.

**Remarks**. We have ten immature individuals collected with an adult. However, we do not presently have the adult to examine, so we cannot identify this taxon to the species level.

**Range**. The genus is previously recorded from Peru. We collected a single aggregation of this gregarious (Legendre et al. 2014) taxon from a single tree at Finca Las Piedras Research Station.

**Habitat**. Low hill forest.

### *Lanxoblatta* Hebard, 1931

#### Lanxoblatta patriciae

Taxon classificationAnimaliaBlattodeaBlaberidae

Vanker, Medina-Espinoza, & Evangelista sp. nov.

C70D2412-B239-5E9F-8FB3-DA228487E4C3

https://zoobank.org/AA956D4B-9552-46A6-9A42-C5966BDEB8E5

Figs 16, 17

##### Type material.

***Holotype***. • Male, pinned, with genitalia in a separate microvial. Original label: “Peru: MD [Madre de Dios], Estación Biológica Los Amigos. Trail 10, terra firme forest, 12°33'59"S, 70°05'59"W [-12.566389, -70.099722], 6.vii. 2021 [6 July 2021], night, hand collected. D.A. Evangelista leg”. AUDE-PE-1-69. ***Paratypes***: • AUDE-PE-1-89 AUDE-PE-1-90, UIRB-PE-26-61, UI RB-PE-26-62, UIRB-PE-26-74, UIRB-PE-26-75, UIRB-PE-26-76, UIRB-PE-26-77, UIRB-PE-26-81, UIRB-PE-26-82, UIRB-PE-26-83 (locality and other data for all specimens are given in Table 3).

**Figure 16.**
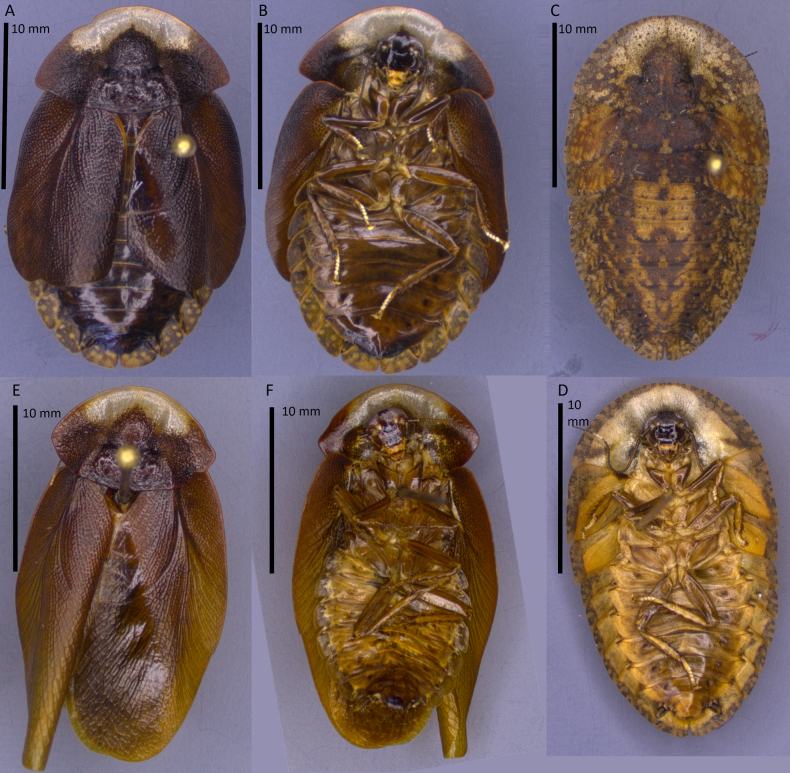
*Lanxoblatta patriciae*. **A**. Dorsal habitus from adult female; **B**. Ventral habitus of adult female; **C**. Dorsal habitus of late-instar immature; **D**. Ventral habitus of late-instar immature; **E**. Dorsal habitus from adult male; **F**. Ventral habitus of adult male. Specimens imaged: (**A, B**) paratype UIRB-PE-26-74, (**C, D**) paratype UIRB-PE-26-77, and (**E, F**) holotype AUDE-PE-1-69. Scale bars: 10 mm (**A–D**).

**Figure 17.**
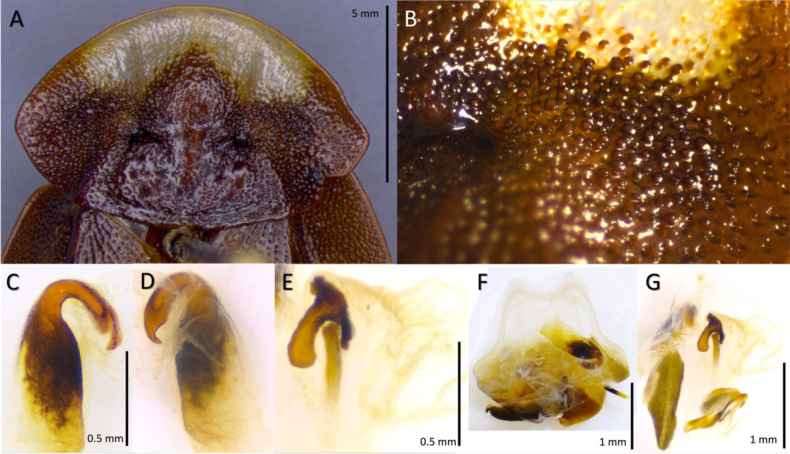
*Lanxoblatta patriciae* adult male morphology, detailed images, holotype (AUDE-PE-1-69). **A**. Pronotum; **B**. Microscopic pronotal warts (30 × magnification); **C, D**. L3’. **E**. *via*. **F**. Subgenital plate with genitalia in place (partially damaged). **G**. All genital sclerites. Scale bars: 5 mm (**A**); 0.5 mm (**C, E**); 1 mm (**F, G**).

##### Determination.

This species was placed in *Lanxoblatta* based on the pronotal morphology (shape, color, and pronotal window shape). We were able to associate juveniles with the adults based on pronotal features.

##### Differential diagnosis.

*Lanxoblatta patriciae*, which is most similar to *L. martinezi* (Bolivar, 1881), and can be separated from all known *Lanxoblatta* based on the body size and pronotal morphology. *L. patriciae* and *L. martinezi* share the feature rugae radiating out from the pronotal center into the pronotal window. *Lanxoblatta patriciae* differs from *L. martinezi* in males being 20 mm long (*L. martinezi* males are 30 mm long; Table 4), and our lone female *L. patriciae* has semi-circular pronotum without any emargination (notch) in it laterally (*L. martinezi* females have an emargination on the pronotum just back of the lateral angles).

##### Remarks.

*Lanxoblatta patriciae* bears impressive pronotal warts, but we are unclear how they compare to pronotal rugosity in other *Lanxoblatta* spp. *Lanxoblatta frater* Hebard, 1933 is described as having “nodules”, and *L. magnifica* Rehn, 1932 bears “tubercules” on the pronotum. *Lanxoblatta patriciae* differs from *L. frater* Hebard, 1933 in having the warts much more extensive throughout the pronotum. We have not examined the tubercules in *L. magnifica* Rehn, 1932 but that species is nearly twice the size of *L. patriciae*. The description of *L. martinezi* (Bolivar, 1881) mentions granules on the pronotum but perhaps the microscopy available at the time was not sufficient to describe their detail more fully.

##### Description of holotype.

**Male** (AUDE-PE-1-69). ***Head***. Two flat ocelli. Antennal scape and pedicel dark brown with paler flagellum. Frons and vertex dark brown, palest between the ocelli. The part of the postclypeus directly under the epistomal suture is paler, this pale area shrinks as the dark coloration, continued from the frons into the postclypeus, expands until reaching the anteclypeus where it abruptly stops, being replaced by a pale coloration. The labrum is banded, going from pale posteriorly, to dark, to pale again towards the mandibles. Maxillary and labial palp darker towards center of each segment, growing paler as the joint is approached.

***Thorax*. *Pronotum***. Roughly elliptical, with a greater distance from the vertexes to the top co-vertex than to the bottom co-vertex. Medial-anterior region of pronotum with a kidney shaped semi-transparent and unpigmented region. Pigmented areas of pronotum are mostly chestnut, with some near black-brown areas. Many small dark warts (protrusions that are narrow at the base and bulbous towards the apex) create a rugose texture. Many of the warts are so protruded that the form pillar-like shapes, only visible at high magnification. The greatest concentration are equidistant from the midline to the edge of the pronotum on either side, growing darker as their density increases, towards the edges of the pronotum the warts transition into shallow indentations. At the center of the pronotum, there are two dark sulci. ***Wings***. Tegmina dark brown. Tegmina thickened basally so minor veins and crossveins are not entirely visible. Instead, there is a roughly uniform network of shallow pits. The subcostal and secondary branching of the radial veins support the laterally broad tegmina. ***Legs***. Forecoxae pale brown. The forefemur is pale brown with a row of setae on ventro-anterior margin, with no apical spine or genicular spine. Foretibia with six spines on the dorsal side, one being smaller than the others, and five large ventral spines on the distal side of a dense patch of setae. Foretarsus composed of five segments, the first four having euplantulae and being significantly smaller than the pre-tarsus. Arolium present, claws simple and symmetrical. Mid femur pale brown with a few thin hairs on ventro-anterior margin. Midtibia pale brown with nine large ventral spines and seven dorsal. Midtarsus is broken but has at least four remaining tarsomeres, first tarsomere longer than its equivalent on the foreleg and the other three tarsomeres on mid tarsus with euplantulae present.

***Abdomen***. The dorsal abdomen is brown, paler than the pronotum but darker than the ventral abdomen. No visible tergal gland present. SA plate damaged in type. Posterior edge of sub-genital plate convexly arcuate with left stylus in a shallow and moderately narrow invagination. Right stylus missing due to damage (present in other specimens).

***Genitalia***. L3’ hooked (see Fig. 17), *lve* long and slender, *via*: flat and broad at base with bulbous protrusion curving upwards to *lve* (see Fig. 17). R phallomere: curving on itself almost in a spiral pattern (see Fig. 17).

Morphological measurements are presented in Table 4.

##### Description of paratype.

**Female**. UIRB-PE-26-62. Head identical to description of male. Pronotum wider than in male, and more acutely angled laterally. Texture and color of pronotum indistinguishable from the male. Tegmina similar to male but truncated, only reaching to the posterior edge of T5. Forelegs like male except only three spines in the dense patch of setae on the ventral tibia, and six spines on the dorsal side. Abdominal flanges more pronounced than in males, with some mottled coloration near the edges. SA plate also more expanded than male, extending past the ends of the cerci, which are also shorter in the female.

##### Range.

This is the first published record of *Lanxoblatta* in Peru. *L. patriciae* is widespread in Madre de Dios, but we did not find it at the two suburban sites near Puerto Maldonado. We hypothesize that it may be part of an evolutionary grade (parapatry) with *L. martinezi* because of the similarity with that species. This could suggest that its range to extends northward throughout the Peruvian Amazon.

##### Habitat.

Low hill forest and flooded alluvial forest.

##### Etymology.

The specific epithet is in honor of the grandmother of KV.

### Blaberinae Saussure, 1864

***Blaberus* Serville, 1831**

#### *Blaberus* sp.

**Material**. AUDE-PE-1-93, AUDE-PE-1-94, AUDE-PE-1-97, AUDE-PE-1-98, UIRB-PE-20-01, UIRB-PE-20-02, UIRB-PE-20-03, UIRB-PE-20-04, UIRB-PE-20-05, UIRB-PE-20-06, UIRB-PE-20-07, UIRB-PE-20-08, UIRB-PE-20-09, UIRB-PE-20-10, UIRB-PE-20-12, UIRB-PE-20-13, UIRB-PE-20-14, UIRB-PE-20-15, UIRB-PE-20-16, UIRB-PE-20-17, UIRB-PE-20-18, UIRB-PE-20-21, UIRB-PE-20-22, UIRB-PE-20-23, UIRB-PE-20-24, UIRB-PE-20-25, UIRB-PE-20-26, UIRB-PE-20-27, UIRB-PE-20-28, UIRB-PE-20-29, UIRB-PE-20-30, UIRB-PE-20-31, UIRB-PE-20-32, UIRB-PE-20-34, UIRB-PE-20-36, UIRB-PE-20-46, UIRB-PE-20-47, UIRB-PE-20-48 (locality and other data for all specimens are given in Table 3).

**Determination**. Four adult specimens were identified as *Blaberus* based on the huge size and distinctive color pattern (nearly all white tegmina and pronotum except for the death-head pronotal spot). Thirty-four immature individuals were also identified based on their huge size and distinctive color patterns common to members of the genus. We could not determine these to species level because of the low differentiation of the genitalia (Roth 1969).

**Range**. While this was the most abundant blaberid collected, many individuals were collected from aggregations in just a handful of trees and logs.

**Habitat**. Low hill forest and flooded alluvial forest.

### Hormeticini Burmeister, 1838

#### Hormeticini sp.

**Material**. UIRB-PE-21-20, UIRB-PE-21-04, UIRB-PE-29-40, UIRB-PE-20-69 (locality and other data for all specimens are given in Table 3).

**Determination**. These immature individuals are assumed to be Blaberinae based on the large body size, stout cerci, and robust form.

**Remarks**. These immature individuals, which like much larger versions of immature *P. surinamensis*, are unlikely to be associated with any other taxon referenced here. We list them here as an additional species in the region. We guess that these are *Oxycercus* or another Hormeticini sp.

**Range**. We only recorded this taxon from Kawsay Biological Station.

**Habitat**. Flooded alluvial forest.

#### *Phoetalia* Stål, 1874

**History**. The most rigorous morphological treatment to date (Roth 1970c) placed the genus in Brachycolini Rehn, 1951 based on the characters: *via* not solidly attached to *lve* and separated from it by a membrane, and fringe of preputial spines on *via*. Prior, the genus had been placed by Princis (1960) and Hebard (1917) in Epilamprinae and by McKittrick (1964) in Diplopterinae.

The genus has been included in a number of molecular phylogenies. While the relationships among Blaberinae and Zetoborinae genera are quite volatile, most multi-gene studies place *Phoetalia* as sister to *Schultesia* (Legendre et al. 2017; Bourguignon et al. 2018; Djernæs et al. 2020), or as sister to a clade of Zetoborinae containing *Schultesia* (Legendre et al. 2014). Phylogenomic studies place *Phoetalia* in Blaberinae, although distant from Hormeticini (Evangelista et al. 2020; Evangelista et al. 2024; Deng et al. 2026).

The multi-gene studies, which are largely based on mitochondrial DNA data (Legendre et al. 2017; Bourguignon et al. 2018; Djernæs et al. 2020), agree somewhat with Roth (1970c)’s determination. These studies mostly recover Hormeticini as sister to the clade containing *Phoetalia*. In this interpretation, the synapomorphies identified by Roth (1970c) would be symplesiomorphies. Studies by Evangelista et al. (2020); Evangelista et al. (2024); and Deng et al. (2026) are less congruent with Roth (1970c)’s morphological interpretation. The specimen sequenced by Evangelista et al. (2020), which was then used in their subsequent studies, was taken from a population in a live culture. It is possible a juvenile was sampled and could have been misidentified. This would explain the discrepancy in placement of samples between the studies. If future sequencing of genome-scale data from *Phoetalia* spp. corroborates the relationship with *Schultesia*, then perhaps the placement could be considered resolved.

##### Phoetalia amigo

Taxon classificationAnimaliaBlattodeaBlaberidae

Vanker, Medina-Espinoza, & Evangelista sp. nov.

B154E197-A18B-5C9B-B7AA-B3948D8471CE

https://zoobank.org/155C2584-1059-4CCA-B11F-842A39BA14FA

Figs 18, 19

###### Type material.

***Holotype***. • Male, pinned, with genitalia in a separate microvial. Original label: “Peru: MD [Madre de Dios], Estación Biológica Los Amigos. In/around buildings, terra firme, 12°34'08.9"S, 70°06'01.3"W [-12.569139, -70.100361]; 5–8.vii.2021 [5–8 July 2021], night. D.A. Evangelista leg”. AUDE-PE-1-95. ***Paratype***. • Female. UIRB-PE-30-77 (locality and other data for both specimens are given in Table 3).

**Figure 18.**
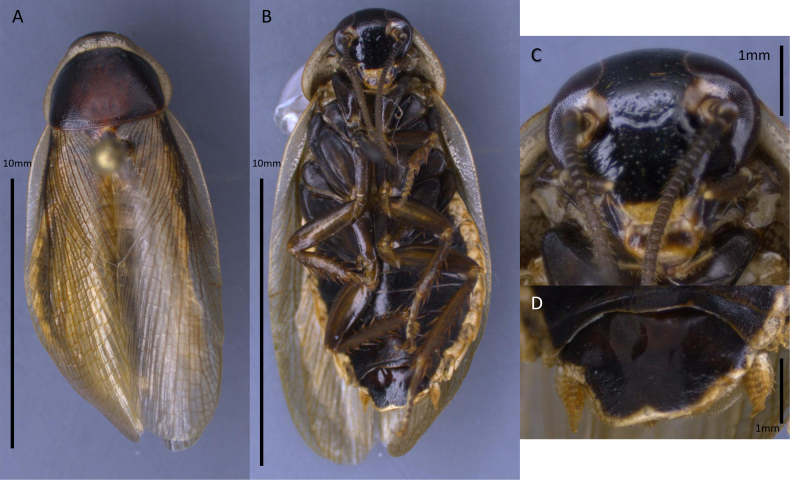
*Phoetalia amigo* sp. nov. adult male, holotype (AUDE-PE-1-95). **A**. Dorsal habitus; **B**. Ventral habitus; **C**. Details of the head; **D**. Sub-genital plate. Scale bars: 10 mm (**A, B**); 1 mm (**C, D**).

**Figure 19.**
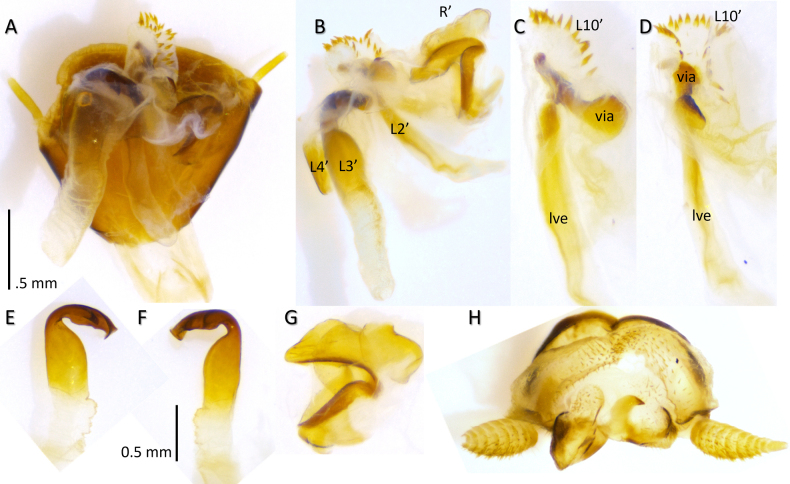
*Phoetalia amigo* sp. nov. male sexual morphology, holotype (AUDE-PE-1-95). **A**. Dorsal view of sub-genital plate with genitalia in place; **B**. Genitalia without sub-genital plate; **C**. View of L2’ showing highly elongated via; **D**. L2’; **E**. Dorsal view of L3’; **F**. ventral view of L3’; **G**. R’ phallomere from dorsal view; **H**. Supra-anal plate (ventral) and paraprocts (posterior). Scale bars: 0.5 mm (**A, F**).

###### Determination.

This species was placed in Blaberinae based on the presence of sclerite L4’ (Klass 1997) and by the large crown of spines on L10’. We tentatively placed it in *Phoetalia* after comparisons of genitalia in *Phoetalia* (L10’ spines present; (Roth 1970c), *Parasphaeria* (L10’ spines absent), and *Schultesia* (L10’ spines absent and has a differently shaped *via*; (Roth 1973). Grandcolas and Pellens (2002) mentioned the left paraproct forming a blunt, curved hook as a synapomorphy of *Parasphaeria* but it is also present in *P. amigo*.

###### Differential diagnosis.

*Parasphaeria amigo* differs from *P. circumvagans* (Burmeister, 1838) and *P. pallida* (Brunner von Wattenwyl, 1865) in the shape of *via*, which is highly elongated in *P. amigo*. *P. amigo* and *P. circumvagans* are similar in pronotal coloration, which both have a pale boarder with a uniform width on the anterior-lateral margin (vs *P. pallida* in which the interface between the pale and dark regions is sinusoidal).

###### Remarks.

Grandcolas and Pellens (2002) mentioned the left paraproct forming a blunt, curved hook as a synapomorphy of *Parasphaeria* but it is also present in *P. amigo*.

###### Description of holotype.

**Male** (AUDE-PE-1-95). ***Pronotum***. Sub-semicircular, with no pigmentation on anterior-lateral margin. Central mahogany-color region reaches posterior margin. Tegmina extends past end of abdomen, and mostly lacks pigmentation except for brown highlights on the veins, which is more prominent basally. Head with frons uniformly black smooth, with slight texture and sparse dimples throughout. A slight concavity connecting the ocelli create a slight W shape (Fig. 18C). Ocelli white without a defined border, placed next to the superior inner part of the antenna. Clypeus and labrum paler than remainder of head. ***Ventral body***. Almost uniformly dark chestnut colored, abdomen bordered by buff all around. Foreleg femur with no genicular spines, and AV margin spination type B1 with one large basal spine and 20–24 small hairs/spinules. Legs with euplantulae on all legs and arolia on middle and hind legs (foreleg tarsus broken). ***Genitalia***. SA plate with a patch of hairs, densest medially. Paraprocts asymmetrical, left paraproct forming a stout, slightly curved hook. Right paraproct amorphous. SG plate slightly asymmetrical, with slightly more concave margin on right. Base of subgenital plate long and without pigmentation. Right stylus short and projecting from the nadir of the concavity. Left style projects from left corner of subgenital plate. Styli otherwise symmetrical. Morphological measurements are presented in Table 4.

###### Description of paratype.

**Female** (UIRB-PE-30-77). Same as male except larger, and with the following departures. Subtle concavity between ocelli is U-shaped. Tegmina only reaching to slightly before SA plate. Foreleg AV margin type B1 with two basal spines. Arolia on fore and mid-legs (hindlegs broken) and euplantulae on all tarsomeres (likely same as male).

###### Range.

We do not have any data on this species outside of Madre De Dios, Peru. We collected only a single adult male of this species at Los Amigos Biological Station. The female described above is from Kawsay Biological station. We have numerous immatures (all from Finca Las Piedras) tentatively identified to *Phoetalia*, and they appear more like *P. amigo* than *P. pallida*.

###### Habitat.

Low hill forest.

###### Etymology.

The specific epithet *amigo*, Spanish for friend (masculine) is a reference to the collection area of the male type, Los Amigos Biological Station.

#### *Phoetalia pallida* (Brunner von Wattenwyl, 1865)

**Material**. UIRB-PE-30-76 (Male) (locality and other data for all specimens are given in Table 3).

**Determination**. This specimen was determined to be *Phoetalia* based on the color pattern of the pronotum and general look of the body, which was then confirmed by comparison of male genitalia.

**Remarks**. This specimen differs slightly from *P. pallida* imaged by Roth (1970c), in the larger, more robust spines on L10’, and also differs from specimens illustrated by Lopes (2004) in the frons coloration (in our specimen, almost entirely black except for pale clypeus and the part of the frons below the antennal scrobes). However, given the large range and documented polymorphism in this species complex (Lopes, 2004), we tentatively assign the specimens to the identification of this same species.

**Range**. This species was previously reported from Peru (Shelford 1912).

**Habitat**. Flooded alluvial forest.

### *Hyporhicnoda* Hebard, 1920

#### *Hyporhicnoda humilior* Hebard, 1933

Fig. 20

**Material**. AUDE-PE-1-70, AUDE-PE-1-91, AUDE-PE-1-92 (locality and other data for all specimens are given in Table 3).

**Figure 20.**
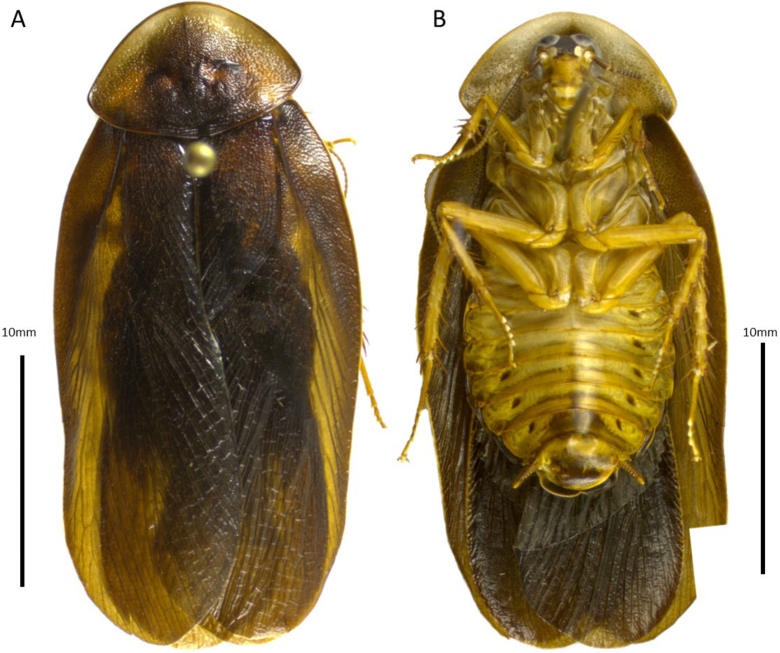
*Hyporhicnoda humilior* Heard, 1933 adult male, AUDE-PE-1-70. **A**. Dorsal habitas; **B**. Ventral habitus. Scale bars: 10 mm (**A, B**).

**Determination**. The adult specimen was identified as Blaberinae based on general similarity to other taxa (e.g., *Blaptica*, *Hyporhicnoda*) and lack of similarity to other South American Blaberidae. The specimen was practically identical to the original description of *H. humilior* and differed from all other *Hyporhicnoda* spp. by the pronotal shape (not-reflexed, medial-longitudinal carina present), general coloration, and glossy surface of tegmina (vs dull).

**Remarks**. This is the first record of *H. humilior* in South America, which was previously only known from Panama. Within Madre De Dios, we only collected this species at Los Amigos Biological Station.

**Habitat**. Low hill forest.

### Panchlorinae Burmeister, 1838

***Achroblatta* Saussure, 1893**

#### *Achroblatta luteola* (Blanchard, 1843)

Fig. 21A, B

**Material**. AUDE-PE-2-92, AUDE-PE-2-97, AUDE-PE-2-99 (locality and other data for all specimens are given in Table 3).

**Figure 21.**
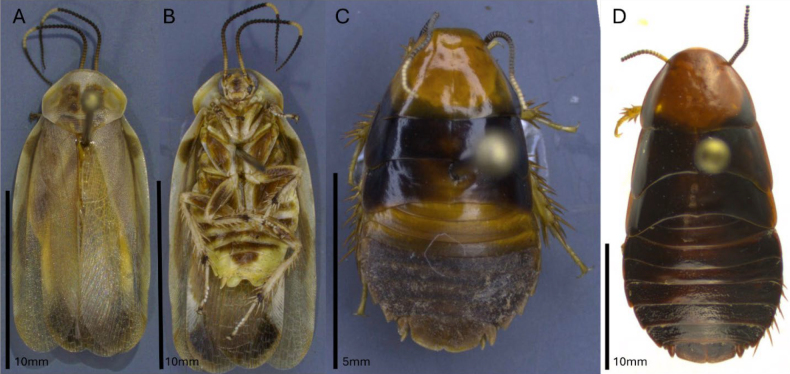
Some Panchlorinae of Madre De Dios. **A** Dorsal habitus of adult male *Achroblatta luteola* (Blanchard, 1843); **B** Ventral habitus of adult male *Achroblatta luteola* (Blanchard, 1843). **C, D**; Possible immatures of Panchlorinae spp., Specimen AUDE-PE-2-95 (**C**) is an earlier instar than AUDE-PE-1-71 (**D**). Specimens imaged: (**A, B**) AUDE-PE-2-92, (**C**) AUDE-PE-2-95, and (**D**) AUDE-PE-1-71. Scale bars: 10 mm (**A, B, D**); 5 mm (**C**).

**Determination**. These adult individuals were identified based on unique color patterns of the pronotum, and tegmina, as well as the distinctively bilobed SA plate (Estrada-Álvarez et al. 2023).

**Range**. This is the first published record of this species in Peru. Within Madre De Dios, we only collected this species at Los Amigos Biological Station.

**Habitat**. Low hill forest.

### *Panchlora* Burmeister, 1838

#### *Panchlora* spp.

**Material**. AUDE-PE-3-60, AUDE-PE-3-62, AUDE-PE-3-66, AUDE-PE-3-67, AUDE-PE-3-71, AUDE-PE-3-73, AUDE-PE-3-82, AUDE-PE-3-83, AUDE-PE-3-84, AUDE-PE-3-85, AUDE-PE-3-88, AUDE-PE-3-93, AUDE-PE-3-95, AUDE-PE-4-58, AUDE-PE-4-59, AUDE-PE-4-60, AUDE-PE-4-61, AUDE-PE-4-62, AUDE-PE-4-63, AUDE-PE-9-51, AUDE-PE-9-52, AUDE-PE-9-53, AUDE-PE-9-54, AUDE-PE-9-55, AUDE-PE-9-56, AUDE-PE-9-58, AUDE-PE-9-59, AUDE-PE-9-60, UIRB-PE-33-63 (locality and other data for all specimens are given in Table 3).

**Determination**. These individuals were identified as Blaberidae based on the stout cerci. They are placed in *Panchlora* based on the lightly pigmented and delicate sclera with green coloration. While body coloration is normally a superficial character, it is likely a synapomorphy of Panchlorinae since Neotropical *Panchlora* are nested within African *Panchlora* (Evangelista et al. 2024) and species on both continents are green. Similar green pigment in the sclera is not known from any other Neotropical blaberid but is also found in the African genus *Pronauphoeta*. Note that some *Panchlora* spp. are white, blue, red, or have colorful spots but we collected none of these types.

**Remarks**. This assemblage of 32 individuals likely constitutes at least three species on the basis of varying body size, and black spots on the tegmina. However, because of the extreme difficulty in identifying *Panchlora* spp. (most species are defined on the basis of color patterns that fade quickly after death), we have not attempted to identify them.

**Habitat**. Low hill forest and flooded alluvial forest.

#### Panchlorinae spp.

Fig. 21C, D

**Material**. AUDE-PE-1-71, AUDE-PE-2-95, UIRB-PE-25-99, UIRB-PE-31-07 (locality and other data for all specimens are given in Table 3).

**Determination**. These immature specimens were identified as Panchlorinae by comparison with information provided by Gurney and Roth (1972). Namely, the pronotum being more pale than the general body color, the texture of the terminal tergites, the spination of the foreleg femur, and the general appearance.

It is possible that our four specimens, which are of different instars and have variable coloration (Fig. 21C, D), constitute more than one species, or genus. Of the taxa treated by Gurney and Roth (1972), *Anchoblatta*, *Achroblatta*, and *Panchlora* are known from Peru. One iNaturalist observation tentatively identified as *Pelloblatta* (https://www.inaturalist.org/observations/163665220) appears almost identical to one of our specimens (Fig. 21D) but this genus is only known from Central America so far.

**Habitat**. Low hill forest.

## Discussion

Combining our novel collection data with records from the literature (Hopkins and Beccaloni 2024), iNaturalist, and GBIF, we compile a checklist of Blaberidae from Peru (Table 5), noting which taxa are specifically known from the Madre De Dios region.

**Table 5.** Checklist of Blaberidae species in Peru and in the department of Madre de Dios.

Subfamily	Genus	Species		Present in Peru?^1^	Present in Madre De Dios?^2^	Record type^3^
Blaberinae	* Blaberus *		Serville, 1831	+	+	Lit, iNat, new
Blaberinae	* Blaberus *	*Blaberus parabolicus*	Walker, 1868	+	unk.	Lit
Blaberinae	* Blaberus *	*Blaberus peruvianus*	Jurberg, Albuquerque, Rebordoes, Goncalves & Felippe, 1977	+	unk.	Lit
Blaberinae	* Blaberus *	*Blaberus scutatus*	Saussure & Zehntner, 1894	+	unk.	Lit
Blaberinae	* Blaberus *	*Blaberus valleyanus*	Lopes & Oliveira, 2013	+	unk.	Lit
Blaberinae	* Blaberus *	*Blaberus yuracianus*	Lopes & Oliveira, 2013	+	unk.	Lit
Blaberinae	* Eublaberus *		Eublaberus Hebard, 1920	+	+	iNat
Blaberinae	* Eublaberus *	*Eublaberus posticus*	(Erichson, 1848)	+	unk.	Lit
Blaberinae	* Eublaberus *	*Eublaberus distanti*	(Kirby, 1903)	+	+	iNat
Blaberinae	* Eublaberus *	*Eublaberus marajoara*	Rocha e Silva, 1972	~	~	iNat
Blaberinae		Hormeticini sp.		+	+	new
Blaberinae	* Oxycercus *		Bolívar, 1881	+	unk.	Lit
Blaberinae	* Oxycercus *	*Oxycercus peruvianus*	Bolívar, 1881	+	unk.	Lit
Blaberinae	* Phoetalia *		Stål, 1874	+	+	new
Blaberinae	* Phoetalia *	*Phoetalia pallida*	(Brunner von Wattenwyl, 1865)	+	+	Lit, new
Blaberinae	* Phoetalia *	*Phoetalia amigo*	sp. nov.	+	+	new
Blaberinae	* Hyporhicnoda *		Hebard, 1920	+	+	new
Blaberinae	* Hyporhicnoda *	*Hyporhicnoda humilior*	Hebard, 1933	+	+	new
Blaberinae or Zetoborinae	* Schultesia *		Roth, 1973	+	+	iNat
Blaberinae or Zetoborinae	* Schultesia *	*Schultesia lamprydiformis*	Roth, 1973	+	+	iNat
Epilamprinae	* Epilampra *		Burmeister, 1838	+	+	Lit, iNat, new
Epilamprinae	* Epilampra *	*Epilampra azteca*	Saussure, 1868	+	+	iNat, new
Epilamprinae	* Epilampra *	*Epilampra conferta*	Walker, 1868	+	+	Lit, iNat, new
Epilamprinae	* Epilampra *	*Epilampra conspersa*	Burmeister, 1838	+	+	Lit
Epilamprinae	* Epilampra *	Epilampra sp. cf. hamiltoni ^4^	(Rehn, 1903)	+	+	Lit
Epilamprinae	* Epilampra *	*Epilampra yompori*	sp. nov.	+	+	new
Epilamprinae	* Epilampra *	*Epilampra grisea*	(De Geer, 1773)	+	+	new
Epilamprinae	* Epilampra *	*Epilampra opaca*	Walker, 1868	+	+	new
Epilamprinae	* Epilampra *	*Epilampra homage*	sp. nov.	+	+	new
Epilamprinae	* Epilampra *	*Epilampra wandpero*	sp. nov.	+	+	new
Epilamprinae	* Epilampra *	*Epilampra sodalis*	Walker, 1868	+	+	new
Epilamprinae	* Epilampra *	*Epilampra paititi*	sp. nov.	+	+	new
Epilamprinae	* Galiblatta *		Hebard, 1926	+	+	iNat, new
Epilamprinae	* Galiblatta *	*Galiblatta* sp.		+	+	iNat, new
Epilamprinae	* Orchidoeca *		Gurney & Roth, 1976	+	unk.	
Epilamprinae	* Orchidoeca *	*Orchidoeca peruvia*	Gurney & Roth, 1976	+	unk.	Lit
Panchlorinae	* Achroblatta *		Saussure, 1893	+	+	iNat, new
Panchlorinae	* Achroblatta *	*Achroblatta luteola*	(Blanchard, 1843)	+	+	iNat, new
Panchlorinae	* Anchoblatta *		Shelford, 1909	+	+	iNat, Lit
Panchlorinae	* Anchoblatta *	*Anchoblatta signifera*	(Scudder, 1875)	+	unk.	Lit
Panchlorinae	* Panchlora *		Burmeister, 1838	+	+	iNat, new
Panchlorinae	* Panchlora *	*Panchlora kozaneki*	Vidlicka, 2016	+	+	iNat
Panchlorinae	* Panchlora *	*Panchlora exoleta*	Burmeister, 1838	+	unk.	Lit
Panchlorinae	* Panchlora *	*Panchlora moxa*	Saussure, 1862	+	unk.	Lit
Panchlorinae	* Panchlora *	*Panchlora peruana*	Saussure, 1864	+	unk.	Lit
Pycnoscelinae	* Proscratea *		Burmeister, 1838	+	~	
Pycnoscelinae	* Proscratea *	*Proscratea peruana*	Saussure, 1862	+	unk.	Lit
Pycnoscelinae	* Pycnoscelus *		Scudder, 1862	+	+	iNat
Pycnoscelinae	* Pycnoscelus *	*Pycnoscelus surinamensis*	(Linnaeus, 1758)	+	+	iNat
Zetoborinae	* Phortioeca *		Saussure, 1862	+	+	iNat, new
Zetoborinae	* Phortioeca *	*Phortioeca andeana*	Rehn, 1928	+	unk.	Lit
Zetoborinae	* Phortioeca *	*Phortioeca peruana*	Saussure, 1862	+	unk.	Lit
Zetoborinae	* Tribonium *		Saussure, 1862	~	~	iNat
Zetoborinae	* Tribonoidea *		Shelford, 1908	+	unk.	Lit
Zetoborinae	* Tribonoidea *	*Tribonoidea oniscosoma*	(Saussure, 1895)	+	unk.	Lit
Zetoborinae	* Lanxoblatta *		Hebard, 1931	+	+	iNat, new
Zetoborinae	* Lanxoblatta *	*Lanxoblatta patriciae*	sp. nov.	+	+	new

1. “+” indicates presence in Peru (not necessarily in the department of Madre De Dios). 2. “+” indicates presence in the Peruvian department of Madre De Dios. “~” indicates probable presence in the region based on nearby observations on iNaturalist. “unk.” indicates that we have not confirmed the presence of this species in the Madre De Dios region. 3. “Lit.” indicates that the country-level presence of this taxon was determined based on historical records (Princis 1963, 1964; Hopkins and Beccaloni 2024). “iNat” indicates that presence in the department of Madre De Dios was found on iNaturalist. “New” indicates that presence in the Madre De Dios region was determined through first-hand physical specimen collection. 4. Shelford (1912) reported *Epilampra* (syn*. Audreia*) *hamiltoni* from a single female. While we trust Shelford for the generic determination (he originally described the genus *Audreia* and was very familiar with Epilamprinae) we doubt his specific determination. This species is brachypterous and is otherwise only known from the island of Cuba.

Despite being among the most charismatic of cockroaches, there is much more work to be done on Blaberidae systematics. Thirteen of the 22 species we observed remain unidentified, reflecting a lack of identification tools for juveniles, adult females, and/or entire genera (e.g., *Panchlora*). Approximately eight of the 22 species we newly observed seem to be previously undescribed species, although there is ambiguity in some cases (e.g., Epilampra sp. cf. azteca, *Epilampra conferta* complex). While only having 36% undescribed taxa may be low compared to some other insect groups (Stork 2018), it is surprisingly high considering the amount of time since other new species were described in some of the genera we treat (e.g., *Epilampra*, *Phoetalia*). Prior to this work, the slowdown of taxonomic activity in these genera could have been taken to mean that perhaps there were few new species to be described but the data presented here demonstrates that this is far from true. The question of how many species of neotropical Blaberidae exist is largely dependent on endemism rates. Of the 39 Blaberidae species from Peru, 78% are only known from Madre De Dios. This high proportion certainly reflects a sampling bias, but it is not clear to what degree. Within Made De Dios, 13 of the 22 species were found from a single site, yet this too could be a sampling artefact as four of these were singletons and four others are known to be more widespread from literature records. Thus, 20–40% of the Blaberidae species observed are endemic to single sites. This rate would likely decrease with greater sampling at each site, and with additional sites.

## Supplementary Material

XML Treatment for Epilampra yompori

XML Treatment for Epilampra homage

XML Treatment for Epilampra wandpero

XML Treatment for Epilampra sofia

XML Treatment for Epilampra paititi

XML Treatment for Lanxoblatta patriciae

XML Treatment for Phoetalia amigo

## Footnotes

See Roth (1970d) for distinguishing among the three subdivisions of this group, which classify a large number of species, including a few of the Peruvian species we report here.

## Additional information

### Artificial Intelligence (AI) use

The authors accept full responsibility for the content of the manuscript, including the disclosure of any use of AI.

No AI tools were used in the preparation of this manuscript.

### Author ORCIDs

E.F. Medina-Espinoza https://orcid.org/0000-0002-9600-9557

D.A. Evangelista https://orcid.org/0000-0003-3910-5099

### Data availability

All of the data that support the findings of this study are available in the main text or Supplementary Information.

## Supplementary materials

Supplementary material 1 Checklist of Blaberidae species in Peru and in the department of Madre de Dios Data type xlsx Explanation note 1. 1. “+” indicates presence in Peru (not necessarily in the department of Madre De Dios).2. 2. “+” indicates presence in the Peruvian department of Madre De Dios. “~” indicates probable presence in the region based on nearby observations on iNaturalist. “unk.” indicates that we have not confirmed the presence of this species in the Madre De Dios region.3. 3 “Lit.” indicates that the country-level presence of this taxon was determined based on historical records (Princis 1963, 1964; Hopkins and Beccaloni 2024). “iNat” indicates that presence in the department of Madre De Dios was found on iNaturalist. “New” indicates that presence in the Madre De Dios region was determined through first-hand physical specimen collection.4. 4. Shelford (1912) reported Epilampra (syn. Audreia) hamiltoni from a single female. While we trust Shelford for the generic determination (he originally described the genus Audreia and was very familiar with Epilamprinae) we doubt his specific determination. This species is brachypterous and is otherwise only known from the island of Cuba. Counts represent the number of species present in that higher taxon known from the area. https://binary.pensoft.net/file/1668118This dataset is made available under the Open Database License (http://opendatacommons.org/licenses/odbl/1.0/). The Open Database License (ODbL) is a license agreement intended to allow users to freely share, modify, and use this Dataset while maintaining this same freedom for others, provided that the original source and author(s) are credited. Katharine Vanker, Emmy Fiorella Medina-Espinoza, Johanna Schwartz, Jared Martin, Dominic A. Evangelista

Supplementary material 2 New blaberidae occurence data in Madre De Dios, Peru Data type csv Explanation note All newly collected Blaberidae occurence data in DarwinCore format. https://binary.pensoft.net/file/1668119This dataset is made available under the Open Database License (http://opendatacommons.org/licenses/odbl/1.0/). The Open Database License (ODbL) is a license agreement intended to allow users to freely share, modify, and use this Dataset while maintaining this same freedom for others, provided that the original source and author(s) are credited. Katharine Vanker, Emmy Fiorella Medina-Espinoza, Johanna Schwartz, Jared Martin, Dominic A. Evangelista
